# Digital Markers for Passive Remote Monitoring of Bipolar Disorder: Systematic Review

**DOI:** 10.2196/94819

**Published:** 2026-08-13

**Authors:** Thomas P Kutcher, Isha Chakraborty, Kristin Kostick-Quenet, Akane Sano, Nidal Moukaddam, Jeffrey A Herron, Wayne K Goodman, Sameer A Sheth, Ashutosh Sabharwal, Nicole R Provenza

**Affiliations:** 1 Department of Electrical & Computer Engineering, Rice University, Houston, TX, United States; 2 Center for Medical Ethics and Health Policy, Baylor College of Medicine, Houston, TX, United States; 3 Menninger Department of Psychiatry and Behavioral Sciences, Baylor College of Medicine, Houston, TX, United States; 4 Department of Neurological Surgery, University of Washington, Seattle, WA, United States; 5 Department of Neurosurgery, Baylor College of Medicine, 1 Baylor Plaza, Houston, TX, 77030, United States, 1 713 798 4696, 1 713 798 3739; 6 Neuroengineering Initiative, Rice University, Houston, TX, United States; 7 Gordon and Mary Cain Pediatric Neurology Research Foundation Laboratories, Jan and Dan Duncan Neurological Research Institute, Texas Children’s Hospital, Houston, TX, United States; 8 Department of Bioengineering, Rice University, Houston, TX, United States

**Keywords:** digital biomarkers, bipolar disorder, wearables, smartphone, mood disorders, remote sensing technology, passive monitoring

## Abstract

**Background:**

Bipolar disorder (BD) features episodic shifts among mania, hypomania, depression, mixed states, and euthymia. Timely detection of mood transitions is difficult due to infrequent clinical touchpoints. Digital health technologies, including wearables and smartphones, offer a unique opportunity to passively and continuously monitor behavior and physiology that could reflect underlying mood dynamics in real-world settings.

**Objective:**

This study aimed to systematically review passively collected digital markers for BD mood states, characterize devices/modalities and analytic approaches, appraise risk of bias, and identify design gaps and priorities for clinical translation.

**Methods:**

Following the PRISMA (Preferred Reporting Items for Systematic Reviews and Meta-Analyses) guidelines, we searched MEDLINE, PsycINFO, Scopus, IEEE Xplore, and ACM Digital Library (February 16, 2026). We included peer-reviewed studies of adults with bipolar I disorder or bipolar II disorder (BDI or BDII) that measured passively collected markers and related them to depressive, manic, hypomanic, mixed, or euthymic states. Studies that relied exclusively on active measures (eg, lab tests and ecological momentary assessment) were excluded. Two independent reviewers screened studies, extracted study characteristics and results, conducted narrative synthesis, and assessed risk of bias.

**Results:**

Of 23,727 records, 57 studies met criteria. Most enrolled ≤50 participants (n=34, 60%) and monitored ≤365 days (n=46, 81%); 11 out of 57 studies (19%) collected data only in the clinic. Eight digital marker domains emerged: physical activity, heart rate (HR), electrodermal activity (EDA), geolocation, smartphone use, light exposure, sleep, and speech. Consistent patterns linked depression to reduced mobility and social interaction, later/variable sleep, and lower daytime light; mania and hypomania were associated with higher and more variable activity, shorter/advanced sleep, and increased communication. Circadian features derived from sleep/activity repeatedly aided prediction. EDA tended to be lower in depression; HR variability findings were mixed across settings and methods. Keyboard and speech features (eg, timing and prosody) showed associations and performed well in classification models. Twenty-one studies used machine learning; several reported strong performance for episode prediction/classification. However, external validation was usually absent, samples were small, monitoring windows were often short relative to episode timescales, clinical labels were infrequent/misaligned, and missingness was rarely modeled despite likely informativeness.

**Conclusions:**

Passive digital markers for BD show promise, with the most robust signals aligning with *DSM-5* (*Diagnostic and Statistical Manual of Mental Disorders* [Fifth Edition]) diagnostic features (sleep-wake patterns, activity, socialization, geolocation, and speech). To move from promise to practice, future studies should adopt longer within-subject monitoring, align label cadence with sensing granularity, standardize features/reporting, preregister analyses, externally validate models, minimize data collection to protect privacy, and expand physiological measurement beyond HR and EDA. These steps are essential to develop reliable, actionable tools for earlier detection and management of BD mood episodes.

## Introduction

Bipolar disorder (BD) is a chronic, recurrent affective disorder marked by wide fluctuations in mood, energy, and activity that affects more than 1% of the world population [1,2]. BD often results in significant functional impairment and poor quality of life, imposing a significant burden on caregivers and society at large. Patients chronically fluctuate among mood states, including depression, mania and/or hypomania, mixed states (eg, irritable mood and elevated energy), and euthymia (stable mood) [3,4].

BD mood state fluctuations can threaten personal well-being. BD is marked by a suicide rate 20 times higher than that of the general population [5], and suicidality most commonly occurs in depressive and mixed states [6]. Additionally, mania and hypomania may lead to impairments in psychosocial function, reckless behavior (eg, excessive spending, promiscuity, and harming others), psychosis, increased risk of depression relapse, and increased caregiver burden. Generally, clinicians become aware of changes in patients’ mood states through direct patient contact via clinic visits or phone calls [7]. Mood fluctuations in BD are extremely difficult to treat as episodes largely occur outside of clinical observation [8]. If the clinician is made aware of acute depression and mania, the patient can then be treated with the appropriate intervention (eg, pharmacological and behavioral) [2]. Taken together, there is an urgent need for new methods to remotely monitor the mood states of patients with BD outside the clinic that may support earlier clinical intervention during a relapse or episode recurrence.

Recently, studies have investigated the potential of using remote monitoring tools to facilitate BD treatment. Thus far, studies leveraging these tools have not yet demonstrated any significant improvement in patient outcomes [9,10]. Self-monitoring approaches have historically been limited by both poor patient insight and the high levels of patient engagement required for data collection, leading to lapses in compliance [11]. Although specific diagnostic criteria of mania and depression (eg, decreased need for sleep in mania; insomnia or hypersomnia in depression) are potentially measurable via wearable devices, evidence reliably linking these remotely collected measures to specific mood states is inconclusive. While remote passive monitoring methods have demonstrated some preliminary promise, a greater understanding of objectively measurable behavioral or physiological signals (digital markers) of BD mood states is necessary to enhance insights beyond what is learned from patient self-reports [7].

Over the past 5 years, several reviews have been published that are relevant to remote monitoring in BD. A 2021 review explored the state of using portable technologies to monitor patients with BD, focusing mainly on methodological approaches and less so on results [12]. Another 2021 review focused on the specific use of smartphone apps to remotely monitor BD symptoms, looking at both active and passive monitoring practices [13]. However, to our knowledge, no reviews have focused on discussing digital markers of BD mood states that can be remotely and passively monitored, nor included discussion of the many recent studies that have implemented emerging machine learning (ML) and AI-based approaches that offer promising potential for monitoring mood states.

The objective of this study was to systematically review the literature to better understand the state of digital markers that can be used for remote passive monitoring of BD. An improved understanding of the existing literature will lead to a better-informed implementation of remote monitoring practices in psychiatry and identify future work required to validate digital markers of BD, thus improving patient outcomes.

## Methods

### Overview

This review followed the PRISMA (Preferred Reporting Items for Systematic Reviews and Meta-Analyses) guidelines, as reported in Checklist 1 [14]. The review protocol was preregistered in the International Prospective Register of Systematic Reviews (PROSPERO; CRD42024607765).

### Inclusion and Exclusion Criteria

Included studies were English-language, peer-reviewed original research studies, including journal articles and conference proceedings, that examined passively collected digital markers associated with manic, hypomanic, depressive, mixed, or euthymic mood states in adults with bipolar I disorder or bipolar II disorder (BDI or BDII). We defined passive measurements as data collected via devices (eg, smartphones and wearables) without requiring active participant input. Eligible studies either (1) evaluated statistical associations between passive digital measures and mood states (ie, correlational analyses), or (2) used passive measures to classify or predict mood states using statistical or ML approaches. Studies were required to report analytic associations or predictive performance metrics. Eligible studies included two categories: (1) primary studies that evaluated mood states within BD cohorts, including depressive episodes identified within diagnostically mixed samples, and (2) contextual studies that differentiated BD from other psychiatric disorders or healthy controls (HC) using passive digital measures. Findings from between-diagnosis studies were synthesized separately from findings on within-BD mood-state monitoring. We excluded studies that relied on active measurements (eg, completing diary entries, performing lab tests, and short supine recordings), and feasibility-only studies that did not examine associations between passive digital measures and mood state. Studies conducted in clinical settings were eligible provided that they evaluated passive sensing modalities with potential for deployment in remote, free-living monitoring.

### Search Strategy

Five electronic databases were searched (MEDLINE, PsycINFO, Scopus, IEEE Xplore, and ACM Digital Library) on February 16, 2026. Search terms included a combination of keywords related to (1) population, (2) digital markers, and (3) monitoring. All search terms are detailed in the Multimedia Appendix 1.

### Study Selection

Article screening was carried out by independent researchers TPK and IC using the online tool Rayyan (Rayyan Systems Inc) [15]. The first step was deduplication, where Rayyan assisted with flagging included studies with similar titles, abstracts, and authors for removal. The second step was title and abstract screening, during which titles and abstracts that did not meet the inclusion criteria were excluded. For the third step, full-text studies were extracted and evaluated against the inclusion criteria. Authors TPK and IC independently carried out all steps of the study selection process and resolved conflicts through discussion when necessary.

### Data Extraction and Analysis

The following variables were extracted from each included study, where available: study identification, main findings, study design, methods (device/app, measurements, and data characteristics), analytic approach (ML vs statistical association), clinical scales and their sampling frequency, and sample characteristics. For predictive studies, we reported the authors’ primary performance metrics (eg, area under the receiver operating characteristic curve [AUROC], accuracy, sensitivity/specificity, and *F*_1_-score), and for associative studies, effect size estimates (eg, β, odds ratios, and e^B^) with CIs or *P* values exactly as provided by authors in Table S1 in Multimedia Appendix 1. Where multiple metrics were given, we prioritized AUROC for classification tasks and standardized effect sizes for associations. Subsequently, included studies were assigned digital marker categories (speech, geolocation, etc) and device categories (smartphones, wearables, etc). Studies that used multiple digital marker categories in multimodal predictive models were included in the “Modeling Using Features that Span Multiple Categories” section. To convey results, a narrative synthesis approach was used, focusing on thematic analysis of digital marker categories, device types, study outcomes, and methodology, while accounting for heterogeneity and potential biases in study design. Additionally, authors TPK and IC independently assessed the risk of bias in all studies. For studies developing or evaluating predictive models, we applied the Prediction Model Risk Of Bias Assessment Tool (PROBAST) [16], which was used to evaluate issues related to study participants, predictors, outcome definitions, and analytic procedures. For nonpredictive observational studies, we used the Newcastle-Ottawa Scale (NOS) [17] to assess methodological quality based on participant selection, comparability of groups, and reliability of outcome measurement. Potential biases and limitations regarding evidence certainty were discussed qualitatively.

## Results

### Search Results

**Figure 1.**
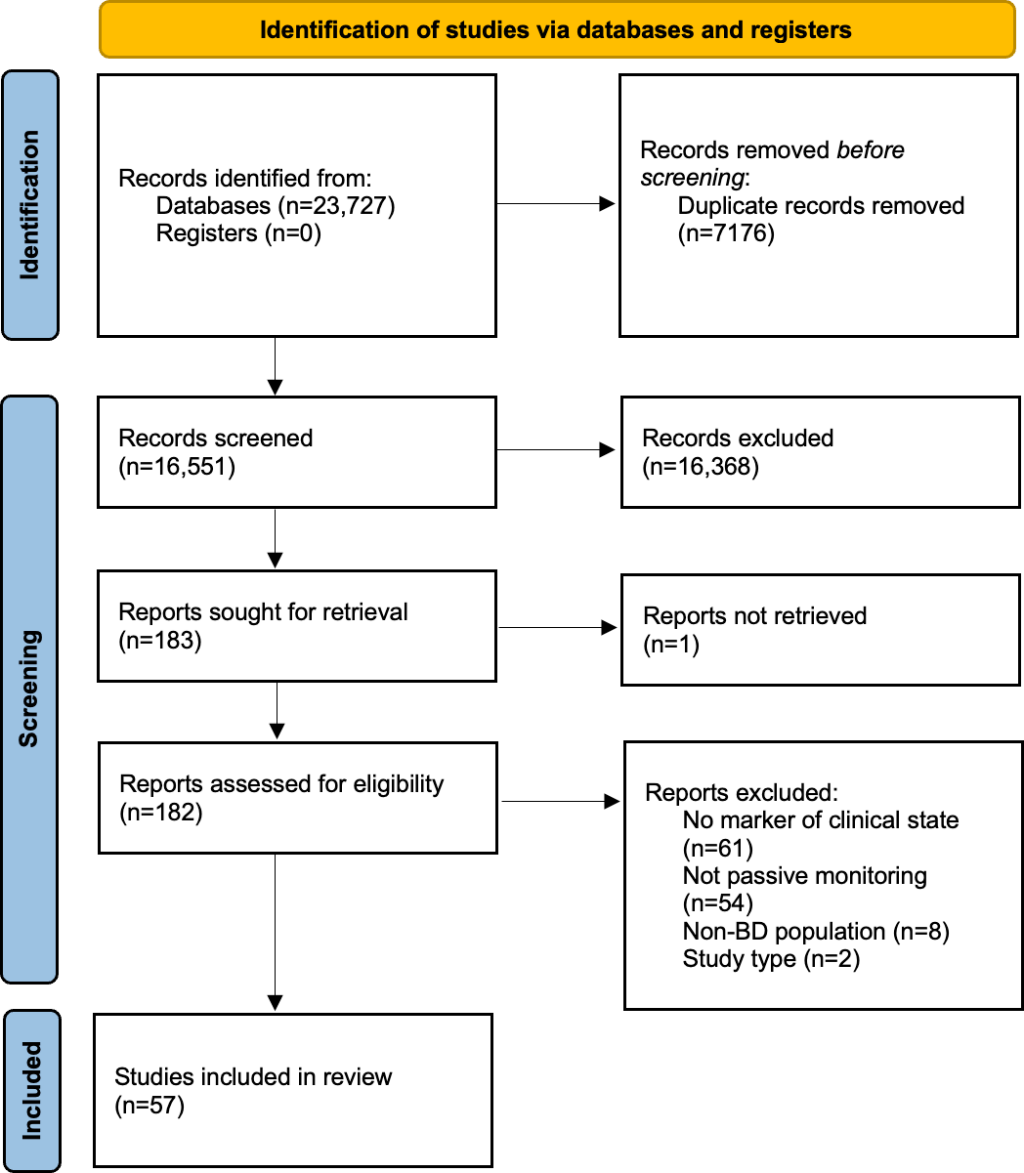
PRISMA (Preferred Reporting Items for Systematic Reviews and Meta-Analyses) study selection flowchart. This flowchart shows the number of articles involved at each stage of the study selection process. BD: bipolar disorder.

The search identified 23,727 records, which were screened as shown in Figure 1. Overall, 57 studies [18-74] were included in the review.

### Study Characteristics

Tables 1 and 2 summarize the characteristics of the 57 studies [18-74] included in this review, with further details in Table S1 in Multimedia Appendix 1. Study sample sizes ranged from 6 to 207 participants, and most studies (n=34, 60%) enrolled 50 or fewer participants. Study designs were heterogeneous, especially in study duration, setting, analysis methods, and clinical scales. Twenty-four [19,21-23,25,32,39,41,42,45,46,48,51,52,54,57-60,62,63,68,70,74] (42%) studies monitored patients for 100 days or less. Eleven [18-21,33-35,48,51,65,70] (19%) studies performed sensor measurements exclusively in a clinical setting. Although these studies were not conducted in free-living environments, they evaluated sensing modalities (eg, wearable sensors or smartphone-based passive sensing) that are potentially deployable for remote monitoring. Some studies relied on between-patient rather than within-patient comparisons. Eight [21,31,39,41,45,52,54,57] (14%) studies captured only acute mood episodes rather than transitions between mood states within the same patient (eg, the transition from mania to euthymia). Only 2 [39,40] included studies did not use a smartphone or wearable device to perform data collection. Nearly all wearable devices used were commercial products, with the exception of 3 [18,34,35] studies from a group that used a custom ECG wearable device. Analytic approaches varied substantially across studies; 21 [20,21,25,34,36-38,43,49,55,56,60,64,67-74] (37%) studies used ML and AI-based methods (eg, supervised classifiers or predictive algorithms), while the remaining studies used traditional statistical association models (eg, correlation analyses, regression modeling, or hypothesis-driven statistical tests) to examine associations between digital markers and mood states. Finally, there was also heterogeneity in clinical scales used to measure mood states. For symptoms of mania or hypomania, 42 [18-24,26,27,31-34,36,37,39,40,44-48,50,53-55,57-64,66-69,71-74] (87.5%) out of the 48 studies that measured mania symptoms used the Young Mania Rating Scale (YMRS). The remaining 6 (12.5%) studies assessed these symptoms using either the Bech-Rafaelsen Mania Scale (BRMS; 3 studies [52,65,70]), the Altman Self-Rating Mania Scale (ASRM; 2 studies [38,49]), or the Structured Clinical Interview for *DSM* (*Diagnostic and Statistical Manual of Mental Disorders*) (SCID; 1 study [28]). Depressive symptoms were assessed using the Hamilton Depression Rating Scale (HDRS) or Montgomery-Åsberg Depression Rating Scale (MADRS) in 40 [18-24,26,27,30-34,37,41-45,47,48,50,52-55,57-64,66,68,69,71,72] studies, while the remaining studies used alternative instruments such as the Quick Inventory of Depressive Symptomatology (QIDS; 5 studies [25,35,36,67,74]), Inventory of Depressive Symptomatology–Clinician Rated (IDS-C; 1 study [46]), Patient Health Questionnaire-8 (PHQ-8; 1 study [38]), Patient Health Questionnaire-9 (PHQ-9; 3 studies [29,49,56]), the Beck Depression Inventory (BDI; 1 study [73]), or the SCID (1 study) [28]. Scales were used at varying frequencies and were subsequently associated with digital markers.

**Table 1.** General characteristics of included studies.

Category	Number of studies (%)
Publication year	
<2014	1 (2)
2014‐2016	11 (19)
2017‐2019	11 (19)
2020‐2022	12 (21)
>2022	22 (39)
Geographic location	
Denmark	11 (19)
United States	8 (14)
Japan	8 (14)
Germany	6 (11)
South Korea	4 (7)
Spain	3 (5)
China	3 (5)
Italy	3 (5)
Poland	2 (4)
Norway	2 (4)
Finland	2 (4)
United Kingdom	2 (4)
Taiwan	2 (4)
Canada	1 (2)
Sample size	
1‐20	16 (28)
21‐50	18 (32)
51‐100	6 (11)
101‐200	15 (26)
>200	2 (4)
Max study duration (days)	
1	1 (2)
2‐7	10 (18)
8‐30	4 (7)
31‐100	9 (16)
101‐365	22 (39)
>365	6 (11)
Not reported	5 (9)
Study setting	
Free-living	46 (81)
Clinical setting	11 (19)

**Table 2.** Sensing and measurement characteristics of included studies.

Category	Number of studies (%)
Sensor type	
Wearable	26 (46)
Smartphone	32 (56)
Photometer	2 (4)
Use multiple sensor types	3 (5)
Wearable type (only wearable sensors)	
Empatica E4	5 (19)
Fitbit	5 (19)
Actiwatch	9 (35)
Custom t-shirt	3 (12)
Oura Ring	1 (4)
Actiheart	1 (4)
Garmin	1 (4)
Movisens	1 (4)
Machine learning or statistical association	
Machine learning	21 (37)
Statistical association	36 (63)
Mania clinical scales used	
YMRS^a^	42 (74)
ASRM^b^	2 (4)
BRMS^c^	3 (5)
SCID^d^	1 (2)
Mania scale not used	9 (16)
Depression clinical scales used	
HDRS^e^	27 (47)
MADRS^f^	13 (23)
QIDS^g^	5 (9)
IDS-C^h^	1 (2)
PHQ-8^i^	1 (2)
PHQ-9^j^	3 (5)
BDI^k^	1 (2)
SCID	1 (2)
Depression scale not used	5 (9)
Measure mood state change	
Yes	49 (86)
No	8 (14)
Measure pre-episode shifts	
Yes	9 (16)
No	48 (84)
Classify diagnostic groups	
Yes	9 (16)
No	48 (84)

a YMRS: Young Mania Rating Scale.

b ASRM: Altman Self-Rating Mania Scale.

c BRMS: Bech-Rafaelsen Mania Scale.

d SCID: Structured Clinical Interview for *DSM* Disorders.

e HDRS: Hamilton Depression Rating Scale.

f MADRS: Montgomery-Åsberg Depression Rating Scale.

g QIDS: Quick Inventory of Depressive Symptomatology.

h IDS-C: Inventory of Depressive Symptomatology–Clinician Rated.

i PHQ-8: Patient Health Questionnaire-8.

j PHQ-9: Patient Health Questionnaire-9.

k BDI: Beck Depression Inventory.

### Passive Digital Markers Cluster Into 8 Distinct Categories

#### Overview

Overall, we divided the digital markers of mood states detailed by the studies included in this review into 8 distinct categories, as detailed in Table 3, according to standard methods used for measurement. Below, we discuss each of the categories, focusing on the methods used to define the digital markers and the significance of the digital markers as established through significant correlations with mood states or importance in predictive classification algorithms. For studies that used multiple feature categories in classification algorithms, we discuss the most important features for accurate mood state classification in this section and elaborate on the methods in the section Modeling Using Features that Span Multiple Categories. Details of individual studies are summarized in Table S1 in Multimedia Appendix 1.

**Table 3.** Categories of digital markers of bipolar disorder mood states.

Marker category	Number of studies (%)	Measurement methods	Example features
Electrodermal activity	3 (5)	Wearable sensors	Mean electrodermal activity (EDA), EDA peaks per minute, and EDA peaks mean amplitude
Geolocation	13 (23)	Smartphone GPS, cell tower, or Wi-Fi	Change in smartphone cell tower ID, change in smartphone Wi-Fi, and GPS
Heart rate	11 (19)	Wearable sensors	Mean heart rate, heart rate variability (HRV), and root-mean-square of HRV
Smartphone usage	15 (26)	Smartphone applications	App usage, screen time; keyboard backspace rate, typing speed, number of incoming and outgoing calls, and texts.
Light exposure	7 (12)	Smartphone (or external) photometer	Average daytime/nighttime illuminance (lux)
Physical activity	21 (37)	Wearable sensors	Acceleration, energy expenditure, and metabolic equivalents (METs)
Sleep	10 (18)	Wearable or smartphone sensors	Total sleep time, sleep efficiency, and sleep variability
Speech	9 (16)	Smartphone (or external) microphone	Speech latencies, pitch, variance, and amplitude

#### Electrodermal Activity

Electrodermal activity (EDA) reflects autonomic nervous system function through changes in skin conductance resulting from sweat gland activity. Three [19-21] studies used wearable devices to establish EDA digital markers. In an in-clinic study recording EDA for 48 hours at inpatient admission and remission, Anmella et al [19] reported lower mean EDA levels and fewer EDA peaks per minute during bipolar depressive states compared to mania and euthymia, with metrics returning to higher levels postclinical remission. Anmella et al and Côté-Allard et al [20,21] identified reduced mean EDA and peak frequency (captured by the Empatica E4 [Empatica Inc] wearable) as important digital markers for bipolar depressive state classification in an acute clinical setting. These findings suggest that EDA may serve as a relevant physiological digital marker, particularly sensitive for depressive episodes, though all 3 [19-21] studies were performed acutely in inpatients. Further work is needed to determine the effectiveness of EDA as a marker in outpatient settings over long periods and state changes within patients.

#### Geolocation

Geolocation, included in 13 [22-31,56,63,74] studies, was measured using changes in smartphone GPS, cell tower, or Wi-Fi. Three [25,56,74] studies used geolocation in multifeature classification models.

Some studies monitored mood states longitudinally in outpatients with smartphones for at least 3 months. Faurholt-Jepsen et al [22,23] found that depressive symptoms were associated with fewer cell tower transitions while manic symptoms were associated with increased cell tower transitions. Extending this finding, Faurholt-Jepsen et al [24] determined that patients with depression moved significantly less per day, spent more time at individual stops, and had less location entropy compared to euthymia. Palmius et al [25] used 10 geolocation-based digital markers across varying timescales to predict QIDS scores using linear regression and classified depressive episodes with a median *F*_1_-score of 0.857. Beiwinkel et al [26] found that reduced mobility (inferred by fewer smartphone cell tower movements) correlated with both depression and mania in 13 patients outside the clinic. In a one-year study, Ludwig et al [27] found significant differences between geolocation metrics (time in vehicle, kilometers traveled) during euthymia compared with the 1‐2 weeks before onset of a depressive or manic episode. In a separate analysis, Ludwig et al [28] found that geolocation features had the strongest associations with hypomania, where distance traveled at speeds >20 km/h (derived from GPS) distinguished prehypomanic and hypomanic periods from euthymia. Effects were inconsistent across other geolocation metrics and time windows. In summary, these studies indicate that a reduction in geographic movements may be a consistent digital marker of bipolar depression. However, the evidence linking these digital markers with mania is mixed.

Geolocation is also useful for predicting depressive states across disorders and differentiating BD from major depressive disorder (MDD). In a large longitudinal study, Aledavood et al [29] found that location variance was lower among depressed patients compared to HC across mood disorders. In another large study, Faurholt-Jepsen et al [30] demonstrated that during depressive states, patients with BD exhibited lower mobility (eg, total duration of moves) compared to patients with unipolar depression. Lee et al [31] found patients with BD to have stronger periodic mobility patterns, higher location variance, and higher entropy compared to MDD.

#### Heart Rate

Heart rate (HR) and HR variability (HRV) were analyzed in 11 [18,20,32-38,43,73] studies using wearable sensors. Faurholt-Jepsen et al [32] reported significantly higher HRV during manic states compared to depressive and euthymic states during a 3-day study in outpatients. Corponi et al [33] found strong evidence for positive changes in HRV as a marker of symptom resolution regardless of depression or mania in inpatients.

Three [18,34,35] small cohort inpatient studies used a custom wearable shirt with integrated electrodes for ECG data collection. Valenza et al [18] demonstrated the feasibility of HRV-based mood state recognition by developing personalized Markov chain models from HRV data collected over 18 hours with a mood state classification accuracy of up to 95.8%. Building on this work, Valenza et al [34] developed support vector ML models for next-day mood forecasting (euthymia vs noneuthymia), achieving prediction accuracies as high as 83.3% (69% on average). Nardelli et al [35] observed reduced HR pattern complexity as measured by the entropy of the R-R interval series in both depressive and hypomanic states.

Six [20,21,36-38,73] studies found that HR-based digital markers were important features in mood state classification algorithms. Notably, in a large longitudinal study, Lipschitz et al [38] found HR to be the most important predictor of hypomania (AUROC=0.852) and among the top predictors of depression (AUROC=0.860) using the Fitbit Inspire (Google LLC) in real-world contexts. Overall, HR and HRV show promise as digital markers for BD mood states relative to euthymia, though findings on HRV vary by methodology.

#### Light Exposure

Seven [36,37,39-43] studies investigated the relationship between light exposure patterns and mood episodes. Four [39-42] of these studies were part of the Association between the Pathology of Bipolar Disorder and Light Exposure in Daily Life (APPLE) cohort based in Japan. In these studies, over 100 patients with BD were monitored for 7 days with photometers in the bedroom and wearables (ActiGraph), and were then followed up at 1 and 2 years [39]. Esaki et al [39,40] found that nighttime bedroom light exposure was significantly associated with current and future manic episodes. Specifically, Esaki et al [39] found that patients with an average nighttime light exposure of ≥3 lux had a higher prevalence of a hypomanic state, whereas Esaki et al [40] used a Cox proportional-hazards model to find that the probability for manic or hypomanic episode relapse was significantly higher when the average nighttime illuminance was ≥3 lux.

Additionally, Esaki et al [41,42] found that higher daytime light intensity was linked to lower depression severity and a lower likelihood of depression relapse. Specifically, Esaki et al [41] found that the highest tertile group in average daytime light intensity had a significantly lower odds ratio for a depressed state than the lowest tertile group. Esaki et al [42] found that a higher average illuminance and longer time >1000 lux during daytime exhibited a significant decrease in relapse into depressive episodes.

Three [36,37,43] large longitudinal studies from the Mood Disorder Cohort Research Consortium (MDCRC) [75] based in Korea supported prior results by determining light exposure during nighttime and daytime as measured by smartphone photometers to be one of the most important digital markers for classifying both manic and depressive episodes. As such, deviations in light exposure patterns may offer an indication of both current and future manic and depressive mood states.

#### Physical Activity

Twenty-one [20,21,27-29,36-38,43-52,56,63,73] studies used wearable sensors to measure physical activity levels through accelerometry. Ebner-Priemer et al [44] found that daily fluctuations in mood were best predicted by changes in same-day activity. Greater activity levels correlated with manic symptoms, whereas depressive symptoms correlated with lower activity as measured by smartphone physical activity measures including GPS travel distances, number of steps, and velocity of movement. Faurholt‐Jepsen et al [45] reported that manic and mixed states have higher energy expenditure compared to euthymia, and higher energy expenditure and trunk acceleration compared to depression. Likewise, Gershon et al [46] found that depressive states were characterized by reduced overall activity and a later activity onset with a midday activity spike and low evening activity. Clemens et al [47] found lower overall activity interdaily stability and a more rigid circadian rhythm in depressed versus euthymic states, while hypomanic states were associated with higher overall activity than euthymia. Additionally, these studies consistently demonstrated that manic episodes were marked by greater overall and more variable physical activity. In a smaller inpatient study, Jakobsen et al [48] showed that manic episodes had greater variability (as defined by sample entropy) in motor activity within-subject compared to euthymia. Ortiz et al [49] found that within-day activity variability achieved high performance in predicting hypomanic episodes with a median time before onset of 2.5 days (IQR 4.0 days). About early state prediction, Ludwig et al [27] in a large longitudinal study found significant differences between physical activity metrics (steps, minutes on foot, and minutes still) during euthymia versus the 1‐2 weeks before onset of a depressive or manic episode. Esaki et al [50] demonstrated that later daily peaks in physical activity rhythms (onset of the 10 consecutive hours with the highest accelerometry amplitude) were significantly associated with increased risk of future depressive relapses.

Nine [20,21,36-38,43,49,56,73] studies found that physical activity-related digital markers were important predictors in multivariable mood state classification algorithms. Notably, 2 [36,37] large longitudinal studies from the MDCRC [75] determined steps at different times of the day, particularly during nighttime, as one of the most important digital markers for classifying both manic and depressive episodes. Additionally, Lipschitz et al [38] found that minutes spent “very active” per day was an important (top 7/17) marker for classification of both depression (AUROC=0.860) and hypomania (AUROC=0.852), while minutes spent sedentary was important only for classification of depression. In smaller studies of acute inpatients wearing Empatica E4 wearable devices, both Côté-Allard et al and Anmella et al [20,21] found acceleration to be one of the most important digital markers in distinguishing mood episodes. In summary, physical activity levels consistently correlate with bipolar mood states; manic episodes are characterized by higher and more variable activity, while depressive episodes show reduced activity and altered timing.

Studies also demonstrate that physical activity can serve as a diagnostic differentiator. In an inpatient study, Tanaka et al [51] highlighted the importance of intraday activity patterns, demonstrating that patients with BD showed morning hyperactivity and evening hypoactivity, whereas patients with MDD showed the opposite pattern. Jones et al [52] found that patients with BD have less stable, more variable, and overall lower circadian activity patterns than controls. Variability in circadian activity patterns was a significant independent predictor of diagnostic group. Aledavood et al [29] found that morning accelerometer variability was negatively associated with depressive symptoms across BD, MDD, borderline personality disorder (BPD), and HC.

#### Sleep

Ten [36-38,43,44,49,53-55,73] studies used wearable sensors to establish sleep digital markers of BD mood states. For example, Ebner-Priemer et al [44] demonstrated that total sleep duration and wake-up time were negatively associated with same-day manic symptoms. In the APPLE cohort, Esaki et al [53] found that variability in total sleep time was strongly associated with future increased mood episode relapse rates, and Esaki et al [54] found a significant association between daytime napping and depressive symptoms. Ortiz et al [49] found that within-night sleep variability achieved high performance in predicting hypomanic episodes and was the earliest indicator of an upcoming hypomanic episode, with a median time before onset of 3 days.

Three [36,37,55] large longitudinal studies from the MDCRC [75] used the Fitbit Charge to measure sleep-related digital markers in over 100 patients with BD outside the clinic. Lim et al [55] used 36 digital markers, all based on sleep and sleep-based circadian rhythms, and extreme gradient boosting (XGBoost) to predict next-day mood episodes in 111 patients with BD (AUROC=0.80, 0.98, and 0.95 for depressive, manic, and hypomanic episodes, respectively). Their study found that circadian phase shifts, as estimated using the sleep/wake time series, were the most significant predictors across depressive, manic, and hypomanic episodes, with delays associated with depressive episodes and advances associated with manic episodes [55]. Cho et al and Lee et al [36,37] determined sleep quality, length, onset deviations, and offset deviations to be important in mania and depression classification. These studies emphasize the predictive utility of sleep patterns and circadian rhythm disruptions (with shorter sleep and advanced rhythms linked to mania, and longer, variable sleep and delayed rhythms linked to depression) in anticipating mood transitions.

#### Smartphone Usage

Fifteen [23,26-29,56-64,74] studies investigated the relationship between smartphone usage and mood states, including keyboard typing, socialization, and other usage metrics. Three [27,28,56] studies captured socialization metrics along with other phone usage metrics. In a series of studies conducted over one year with high compliance, Ludwig et al [27] found significant differences between smartphone usage features in the 1‐2 weeks before depressive or manic episodes compared to euthymia. Specifically, features related to outgoing calls, outgoing not reached calls, unique conversation partners, display on time, and phone inactive time were altered during the 1‐2 weeks leading up to depressive episodes, while only incoming missed calls and outgoing not reached calls were important for distinguishing future manic episodes [27]. In a separate analysis, smartphone socialization features were more informative for depression than for mania, including outgoing calls, incoming missed calls, outgoing not reached calls, and unique conversation partners distinguished selected depressive pre-episode or episode weeks from euthymia, whereas no socialization variables significantly distinguished manic episodes from euthymia [28]. However, effects were inconsistent across variables and time windows [28]. Ikäheimonen et al [56] used an Android smartphone app (Niima; University of Copenhagen) to extract various smartphone usage metrics including app usage, screen time, socialization, and battery levels along with other metrics related to accelerometry and geolocation. They used these metrics as inputs to an ML algorithm (XGBoost) to predict depressive episodes across mood disorders: MDD, MDD with comorbid BPD, and BD. They achieved an *F*_1_-score of 0.82 for predicting depressive episodes, and the 9 most predictive features were related to screen-off time, “leisure” and “social media” app usage, and battery levels at different times of day.

Four [57-60] studies used the BiAffect (University of Illinois Chicago) app on Android smartphones to passively capture keyboard typing metrics [57-60]. In a large study including patients with MDD, Liu et al [57] used a 3-class model of backspace rates (Low, Medium, and High). They found that the Medium group had significantly higher ratings of depression compared to the Low group, and the High group was associated with both nonzero ratings and higher ratings of mania compared to the Low group. In a smaller study, Stange et al [58] found that instability of typing speed (root-mean-square successive difference of each day’s average typing speed) predicted future elevated depression symptoms but not mania symptoms. In another small study, Zulueta et al [59] expanded on this work by examining other keyboard metrics. Using a linear mixed effects model, they found that depression symptoms correlated with greater interkey delay, session count, autocorrect rate, and accelerometer displacement while typing. Using an ordinary least squares model, mania symptoms were correlated with decreased backspace rates and accelerometer displacement while typing [59]. Using the same BiAffect typing dataset, Cao et al [60] applied a deep learning architecture using gated recurrent units to classify bipolar depression with 90% accuracy. Overall, various keyboard metrics, including typing variability, backspace use, and accelerometer displacement, have been associated with both bipolar depressive and manic episodes.

Five [23,26,61-63] studies linked smartphone-based socialization behaviors and mood symptoms in BD. In a longitudinal study of 13 outpatients, Beiwinkel et al [26] found that fewer outgoing texts were predictive of more severe depressive symptoms, while increased outgoing texts were associated with increased manic symptoms. In a larger study, Faurholt‐Jepsen et al [61] observed that a longer duration of incoming calls and a longer duration of outgoing calls were associated with depressive symptoms, while the number of incoming calls, duration of incoming calls, number of outgoing calls, and number of outgoing SMS text messages were associated with manic symptoms. In a separate cohort with additional digital markers, Faurholt‐Jepsen et al [23] observed that during depressive states, individuals exhibited prolonged mobile phone screen-on times, received more incoming calls, were less likely to answer incoming calls, and made fewer outgoing calls [23]. Conversely, during manic episodes, participants sent more outgoing text messages, made longer phone calls, and received shorter text messages [23].

Dominiak et al [62] built upon the results of the Faurholt-Jepsen et al [61] studies and found that depressed patients made fewer phone calls and had a higher fraction of missed calls compared to euthymia, while manic/mixed patients had more outgoing than incoming calls compared to euthymia, though this study had a shorter monitoring period than Faurholt-Jepsen et al [61]. Additionally, in a small study of 10 inpatients, Grünerbl et al [63] found that socialization metrics were an important marker in their multivariable Bayes classification algorithm and achieved an accuracy of 76% in predicting the current mood state using smartphone data and all sensor data. Together, these studies indicate that increased social communication is linked to manic episodes, while reduced communication is characteristic of depressive episodes.

Smartphone usage features were useful in predicting symptoms across disorders and differentiating bipolar depression from unipolar depression. Aledavood et al [29] found that mood disorder patients in depressive episodes (BD, MDD, and BPD) had less consistent phone use rhythms than HC, and depression severity was negatively associated with incoming call duration and positively associated with outgoing call duration in the previous 14 days. Faurholt-Jepsen et al [64] reported that patients with BD in a depressive episode made significantly fewer incoming and outgoing phone calls compared to those with unipolar depression.

#### Speech

Speech was analyzed in 9 [63,65-72] studies using voice recordings from real-world or clinical settings for mood state classification. Zhang et al [65] recorded speech of in-clinic outgoing phone calls using a smartphone app and identified speech digital markers, the fourth formant and linear prediction coefficient, that decreased as patients transitioned from manic to euthymic states, and also found a significant correlation between the linear prediction coefficient and BRMS scores. Another study explored demographic differences in speech-based digital markers using speech during all phone calls for an average of 208 days. Kaczmarek‐Majer et al [66] demonstrated gender differences in speech digital marker associations with mania in terms of volume, pitch, and clarity. Kaczmarek‐Majer et al [66] also found male-specific speech correlations with bipolar depression in terms of volume and clarity, but found no female-specific significant associations. These studies demonstrate that various features of speech may reflect underlying mood states.

In addition to these associations, many studies developed speech classifiers to distinguish mood states with moderate to high accuracy. In 93 inpatients, Ji et al [67] detected depressive and manic states from real-world “journal-style” voice recordings with high accuracy (AUROC=0.861 for depression and 0.903 for mania). In 28 outpatients, Faurholt-Jepsen et al [68] similarly found that voice digital markers from phone calls were highly effective in classifying affective states, with an AUROC of 0.89 for mania and 0.78 for depression. In a small study of 6 outpatients, Karam et al [69] used speech recorded during weekly clinical phone calls and built a classifier for manic (AUROC=0.81) and depressive states (AUROC=0.67). In 21 inpatients, Pan et al [70] differentiated speech by mood episodes in spontaneous speech in conversations with clinicians pre- and postmanic episodes and found that support vector machines performed best for classification within individuals (AUROC=0.886), while Gaussian mixture models were superior for classification across individuals (AUROC=0.727). Together, speech features like pitch and speech latency offer a noninvasive and high-fidelity insight into bipolar mood dynamics, with growing evidence for their diagnostic and predictive validity.

Speech features can also serve as a diagnostic differentiator of BD. In 2 separate studies conducted in over 100 patients over hundreds of days, Faurholt-Jepsen et al [71,72] used naturalistic voice features to distinguish between unipolar and bipolar depression (specificity=0.84; AUROC=0.58), finding that personalized subject models yielded higher performance.

### Modeling Using Features That Span Multiple Categories

Ten [20,21,36-38,43,55,63,73,74] studies used features that span multiple categories of digital markers that were extracted from wearable sensors and/or smartphones to classify mood states. Four [36,37,43,55] studies came from the same group, and each took a slightly different approach toward developing ML models for the classification of mood states based on longitudinal wearable and smartphone data [75]. Across these studies, data were collected continuously for a minimum of 30 days in 37-135 patients with BD, and all analyses used digital markers extracted from the Fitbit Charge wearable device and light exposure digital markers extracted from Samsung smartphones. Cho et al [36] used 130 circadian-rhythm-based digital markers in 37 patients with BD as inputs to random forest models. The models were trained on data from the past 18 days to predict mood episodes in the following 3 days. They reported an AUROC from 0.84 to 0.93 for classifying mood states (euthymia, depression, mania, and hypomania) and identified the most important digital markers as steps during bedtime and daily light exposure for depression, sleep length and sleep quality for mania, and steps during bedtime and daily light exposure for hypomania [36]. Lee et al [37] extended the random forest mood episode prediction analysis to 135 patients with BD and 140 features. They reported AUROC values from 0.95 to 0.98 for predicting depression, mania, and hypomania in the next 3 days. Step counts at bedtime (8-hour window before sunrise) were an important feature for predicting all 3 states [37]. Sleep efficiency was important for depression prediction, while morning light exposure and circadian rhythm acrophase/amplitude were important for mania prediction. In a separate study, Kim et al [43] leveraged circadian phase shifts along with additional wearable-derived features to predict depressive episodes one week in advance across MDD and BD cohorts. Predictive performance (AUROC) was modest (0.565‐0.581), while recall was moderate (0.748‐0.767), consistent with frequent false alarms. Notably, circadian phase shifts, operationalized as dim light melatonin onset, were the strongest predictors of upcoming depressive episodes. Despite relatively modest classification performance, this study uniquely evaluated the longest forecasting window among all included studies. The latest study published by this group used 36 features (all based on sleep and sleep-based circadian rhythms) as inputs to an XGBoost classifier to predict next-day mood episodes in 111 patients with BD [55]. They found that circadian phase shifts (as estimated using the sleep/wake time series) were the most significant predictors across depressive, manic, and hypomanic episodes. Sleep/wake cycle delays were associated with depressive episodes, and advances were associated with manic episodes. The XGBoost classifier yielded AUROC values of 0.925 to 0.985 for predicting next-day depression, mania, and hypomania.

Six [20,21,38,63,73,74] additional studies by other groups also leveraged features that spanned multiple marker categories to predict mood states in BD. Côté-Allard et al [21] proposed a novel deep learning-based ensemble method for euthymia/mania classification in 47 inpatients. The authors used a deep learning model to classify mood states across patients with high accuracy (91.6%) based on 24-hour in-clinic recordings from the Empatica E4. The most important digital markers for accurate classification were acceleration and EDA. Anmella et al [20] performed in-clinic recordings with the Empatica E4 over an extended time period of 48 hours and for repeated time points (acute episode, response, and remission) in 8 inpatients. Using a bidirectional long short-term memory neural network, they achieved 61%‐70% accuracy at classifying episode severity using acceleration, EDA, and HR digital markers. Evidence from Côté-Allard et al and Anmella et al [20,21] converged on acceleration and EDA (via the Empatica E4) as the most important digital markers in distinguishing mood episodes. Grünerbl et al [63] was one of the earliest studies to use multiple categories of features in classifying mood states in real-world outpatients. The authors used digital markers derived from smartphone socialization metrics, phone call speech characteristics, acceleration, and geolocation collected in 10 patients across 12 weeks to classify states with 76% accuracy and a state change detection precision and recall exceeding 97%. A more recent study by Lipschitz et al [38] used the Fitbit Inspire to monitor 17 digital markers relevant to activity and sleep in 54 patients for 9 months. Their study used binary mixed model (BiMM) forest models to classify depression and mania, with AUROC values of 0.86 for depression and 0.852 for mania. The most important digital markers for depression classification were time awake at night, total sleep duration, median bedtime, and resting HR. The most important digital markers for mania classification were HR, sleep efficiency, rapid eye movement (REM) sleep percentage, and active minutes. In another longitudinal study of 24 patients, Wu et al [73] demonstrated that XGBoost with an input of wearable features relating to sleep, physical activity, and HR can predict depressive episodes with an AUROC of 0.89 and an *F*_1_-score of 0.65, and manic episodes with an AUROC of 0.88 but an *F*_1_-score of 0.25. The 6 most important features (12 total) were resting HR, deep sleep duration, floors climbed, average HR, steps, and minimum HR. A large multisite study by Langholm et al [74] demonstrated that multimodal data (geolocation, smartphone usage) could be advantageous in distinguishing between MDD and BD (AUROC=0.61 with support vector machines), and in distinguishing between mood disorders and HC (AUROC=0.91 with random forest).

### Risk of Bias Analysis

#### PROBAST

Twenty-three [16,20,21,25,34,36-38,43,49,51,55,56,60,63,64,67-74] studies were evaluated for the risk of bias in their development of predictive models using PROBAST. While all studies were evaluated to have low risk of bias regarding participants, predictors, and outcomes, all studies demonstrated at least some risk in the analysis domain. This risk was primarily due to the low sample and event sizes and the highly complex multivariate models driving high risk regarding the model’s generalizability to broader populations. Additionally, many models were highly complex and were largely unexplainable in terms of which digital markers were driving classification performance. In addition, none of the studies validated their models on an external dataset, indicating that all studies included are somewhat biased and should be interpreted as preliminary. The complete PROBAST analysis is in Table S2 in Multimedia Appendix 1.

#### The NOS

Thirty-four [17-19,22-24,26-33,35,39-42,44-48,50,52-54,57-59,61,62,65,66] studies were evaluated for risk of bias using the NOS, a widely used tool for assessing the quality of nonrandomized studies. Most studies (n=19) [23,26-29,31,33,39-42,44,46,53,54,61,62,65,66] were rated as high-quality with a low risk of bias as they showed strong methodological rigor in the selection of study groups, comparability, and ascertainment of either exposure or outcome. They typically included well-defined cohorts, appropriate follow-up periods, and robust outcome assessment procedures. However, a smaller subset of the studies (n=15) [18,19,22,24,30,32,35,45,47,48,50,52,57-59] were classified as moderate quality with some risk of bias. These studies often had missing points in the comparability domain, suggesting a lack of control for confounding factors (ie, gender and race), or fewer strengths in outcome ascertainment or follow-up adequacy.. The complete Newcastle-Ottawa assessment is presented in Table S3 in Multimedia Appendix 1.

## Discussion

### Principal Findings

This systematic review identified 57 studies meeting our inclusion criteria that collected objective data from remote monitoring devices (eg, smartphones and wearable sensors) and found associations with BD mood states. Specifically, we found 8 categories of objective digital markers, including physical activity, HR, EDA, geolocation, smartphone usage, light exposure, sleep, and speech. While several digital markers were consistently associated with mood states (eg, decreased geographic movements and smartphone socialization in depression), evidence for others was mixed (eg, HRV in mania). While these studies demonstrate early promise, their heterogeneity in design, duration, and analysis highlights the need for stronger methodological consensus and clinical validation. The converging evidence across studies points toward a future where objective, real-time digital markers may guide early intervention through detection of mood state transitions.

### Diagnostically Grounded vs Exploratory Digital Markers

Five of the digital marker categories we identified are directly grounded in the diagnostic criteria of BD, including geolocation, physical activity, sleep, (smartphone-based) socialization, and speech. These behaviors correspond to the energy and social engagement dimensions of manic and depressive episodes, as specified in the *DSM-5* (*Diagnostic and Statistical Manual of Mental Disorders* [Fifth Edition]) [76]. The *DSM-5* outlines increased goal-directed activity, decreased need for sleep, and heightened sociability as core characteristics of manic episodes, while hypersomnia and social withdrawal are typical of depressive episodes. The alignment of these behaviors with digital markers strengthens their clinical validity. Although some studies refer to these measures as digital biomarkers, we adopt the broader term of digital markers to include both diagnostically grounded measures and more exploratory signals that may capture latent behavioral or physiological processes. For example, manic states were associated with increased activity [23,48], shorter and more frequent phone calls [23], and shifts in sleep patterns [55], while depressive states were accompanied by decreased movement [46], reduced call frequency [62], and increased sleep variability [53]. Additionally, the *DSM-5* recognizes circadian rhythm disruption as a clinically significant marker in BD. Some digital markers in the physical activity, light exposure, and sleep categories can also be classified as circadian rhythm-based digital markers. Specifically, circadian phase and amplitude based on the sleep-wake time series [55], and steps during nighttime [36,37] were identified as important digital markers in deep learning algorithms for mood state classification. Additionally, circadian activity rhythms based on accelerometer measurements have significant associations with mood episode relapses: specifically, robust circadian activity rhythms were associated with a decrease in mood episode relapses, and a later timing was associated with depressive episode relapses [50]. These findings are directly aligned with the understanding that circadian rhythm dysfunction has long been associated with BD [77].

In contrast, digital markers like EDA, HRV, smartphone usage, and light exposure are more exploratory and less tied to *DSM* criteria, but may reflect latent physiological processes underlying mood dysregulation. Specifically, EDA may provide insight into autonomic nervous system functioning and is lower in those with depression [78]. In addition, bedroom light exposure may be an indirect measure of mania [39] and future manic episodes [40], while daytime light exposure intensity may indicate fewer depressive symptoms [41] and episode relapses [42]. Longitudinal studies in larger cohorts will reveal the consistency and generalizability of these exploratory digital markers as predictors of mood state.

### Need for Longitudinal Monitoring

A critical methodological gap identified in this review and reinforced through our bias analyses is the mismatch between the duration of monitoring and the episodic nature of BD. Mood episodes often unfold over weeks to months, yet many studies implemented monitoring windows too brief to capture meaningful mood transitions. For example, studies by Côté-Allard et al and Pan et al [21,70] collected data for less than a month in primarily inpatient environments, limiting the ability to detect the naturalistic dynamics of mood changes. Additionally, for studies that aim to identify markers of hypomanic and mixed states, it is challenging to predict mood states daily when activity is measured every minute, but symptoms are evaluated monthly [46]. In contrast, the most informative and clinically promising studies were those with long-term longitudinal protocols. Included studies with the longest continuous monitoring periods include the BipoSense and MDCRC studies, which monitored participants continuously over 12 months with frequent clinical interviews [44,55]. These studies captured multiple mood episodes and transitions, allowing for the development of temporally rich prediction models. Similarly, the APPLE cohort studies used long-term monitoring to understand circadian rhythm fluctuations [39]. These extended durations allow for robust within-subject modeling, which is critical for identifying pre-episode changes. Future work must prioritize long-term, continuous data collection to enable predictive modeling of transitions into and out of clinically relevant states.

### Frequency of Clinical Labels

To build clinically useful models, the frequency of ground truth labels must align with both the temporal resolution of passive data monitoring and the clinical validity of assessment tools. Although scales commonly administered in these studies, like the YMRS or MADRS, are typically designed to be administered every 2 weeks, many studies chose longer intervals (up to 3 months or longer), compromising the ability to capture transient states. For example, several studies used monthly or even less frequent mood assessments that could miss shorter-lived episodes or rapid cycling [70]. By contrast, some studies administered biweekly clinical scales such as the BipoSense study [27], perhaps striking a better balance between patient burden and temporal precision [22,38]. In the absence of frequent clinical touchpoints, many studies relied on retrospective labeling of mood states using the most recent clinician-administered scale. The retroactive labeling potentially introduces uncertainty when trying to link mood states to passive data on a day-to-day basis, especially when mood fluctuates rapidly or data collection is continuous. Studies should be designed to administer validated clinical scales at recommended intervals to maximize temporal alignment with passive data and enhance the reliability of supervised learning models. Future studies should explicitly define their protocols for labeling ground truth mood states and report how clinician-rated scores are temporally mapped to passive sensing windows, as these decisions critically affect downstream model performance.

### Missing Data and Compliance

Missing data are a pervasive issue and are closely tied to patient compliance and clinical status. In the included studies, there is also a lack of reporting and variability on the handling of missing data and data quality, which is a major concern when it comes to smartphone and wearable data. Few studies explicitly modeled missingness, but several [26,49] acknowledged gaps in wearable device usage. Of studies that reported compliance, the BipoSense studies were among the few that demonstrated excellent compliance rates among participants [44]. Reported compliance rates by study can be found in Table S1 in Multimedia Appendix 1. In our own work in patients with obsessive-compulsive disorder (OCD), we found that patients were more likely to engage in remote monitoring activities (eg, recharging wearable devices and responding to self-reports) when OCD symptoms were minimal, suggesting that lack of compliance may indicate symptom exacerbations [79]. Likewise, nonwear during depressive episodes could signal social withdrawal or fatigue, whereas erratic device usage might suggest mania. Rather than ignoring missing data patterns or treating them as noise, future models may consider integrating missing data as a potentially informative signal for this population. To improve data completeness and clinical utility, it may be best to design passive sensing systems that reduce the need for active participation. Passive sensing would reduce patient burden and bypass states characterized by poor insight when patients cannot identify warning signs in their behavior. Reducing obtrusiveness and invasiveness not only enhances scalability and effectiveness but also supports ethical goals like patient autonomy and promoting ease of use.

### ML Methods for Mood State Classification

The recent emergence of ML and AI methods marks a significant shift toward more sophisticated monitoring practices. This emergence is well-documented, with other recent reviews covering the application of ML and AI-based approaches in detecting mental health states [80,81]. These recent reviews come to a similar conclusion to this review: further work is needed to validate findings. While many studies reported high classification accuracy or AUROC, very few explored the trade-offs between sensitivity and specificity that are critical for patient care. False positives may lead to over-monitoring and increased patient burden, whereas false negatives may delay care. Model optimization should also consider the deployment context and temporal nature of the data collected when recommending an intervention. Common pitfalls included overfitting due to small sample sizes, lack of interpretability, and temporally unaware models. The small sample sizes present in many included studies can also limit the generalizability of ML models given the heterogeneity of BD across patients and mood states. It is possible that models trained on limited datasets may learn cohort-specific patterns that do not generalize across populations. Future work should also prioritize explainable AI (XAI) approaches that improve the transparency and clinical interpretability of predictive models. Streamlining model architectures to reduce model complexity may improve interpretability and clinical trust, while also reducing the potential for overfitting in small cohorts. Post-hoc explanation methods may also help clinicians understand which digital markers are most predictive so that model outputs are more clinically actionable [82]. None of the included studies developed models that spanned more than 4 of the marker categories we identified in this review. In the future, researchers should consider a smartphone and wearable-based approach to passive sensing that integrates the digital markers summarized in this review for improved predictive performance, as wearable-only sensing approaches miss out on important smartphone-based digital markers that have been implicated in BD, such as geolocation and smartphone use.

### Need for Objective Markers to Understand Disease Mechanisms

Psychiatric care has long relied on observed behaviors and self-reported symptoms, which are intermittent, context-dependent, and shaped by underlying biological, psychological, and social factors. While these measures are clinically essential, reliance on behaviors and symptoms limits mechanistic insight and makes it difficult to build objective markers that generalize across patients and settings. In our review, only 2 digital marker categories (HR and EDA) were primarily physiological, while the rest capture behavioral manifestations of illness. This lack of biological and physiological markers is mainly due to the limited capability of widely accessible wearable sensors to capture relevant signals. To move the field toward a biologically grounded understanding of BD, future work should pair continuous behavioral sensing with equally continuous measures of physiology, neural activity, and social context. Practical avenues include emerging biochemical and physiological sensors, including skin patches that sample biochemical signals such as stress hormones or inflammatory markers [83], and recording-capable neuromodulation devices like deep brain stimulation systems used in treatment-resistant psychiatric patients [79,84]. However, biological signals alone are unlikely to fully explain psychiatric states, which are also shaped by interpersonal relationships, stress, environment, and access to care. Prospective studies should collect these streams in the same individuals over extended periods, align them to clearly defined clinical events, and test whether biological, behavioral, and contextual signals can explain clinical states beyond behavior alone. A map linking behavior, physiology, brain activity, and social context would improve our mechanistic understanding of psychiatric disorders and identify actionable targets.

### Digital Markers Across Diagnostic Boundaries

Cross-diagnostic studies are important as they show that passive sensing signals may capture both shared and disorder-specific aspects of pathology. Recent studies by Kim et al and Ikäheimonen et al [43,56] provide evidence that the digital marker categories identified in this review may enable transdiagnostic prediction of depressive episodes across BD, MDD, and even BPD. The convergence of results across BD and MDD cohorts highlights the value of transdiagnostic markers of depression, enabling generalizability in predictive models. This could be useful for model development, especially in small BD cohorts, where pretraining in general mood disorder samples may improve predictive performance. The strong overlap between the predictive domains in these studies and the 8 marker categories discussed in this review suggests that many of these markers capture fundamental behavioral and physiological mechanisms of depression. Therefore, ongoing studies such as Remote Assessment of Disease and Relapse in MDD (RADAR-MDD), the largest study of remote monitoring in mental health, may generalize depression prediction to BD cohorts [85]. On the other hand, several included studies also indicate that passive sensing can help distinguish BD from other groups (MDD and HC). Future studies should further explore digital phenotyping in diverse cohorts to determine similarities and differences in depression prediction across mood disorders.

### Ethical Considerations

The limited validity, accuracy, generalizability, and reliability of existing digital markers for BD and other conditions carry well-recognized ethical ramifications because they directly affect the efficacy and safety of marker-guided clinical decisions. Yet, concerns extend beyond the predictive accuracy of mood states. Efforts to identify objective digital markers for BD also raise serious privacy issues: passively and continuously capturing multiple streams of sensitive data as patients navigate their daily lives can expose intimate details of behavior and physiology [86]. Clinical implementation also raises broader concerns beyond privacy, including the risk that passive monitoring could be used to replace rather than support existing psychiatric care, despite the central role of relationships and face-to-face assessment. Continuous sensing raises the risk of surveillance or coercive use, particularly in settings where psychiatric care can involve involuntary treatment or lack of prohibitions on unwanted data use. Consequently, we advocate a patient-centered data minimization approach that restricts collection to streams with clear, empirically demonstrated clinical value, that is, data shown to meaningfully refine assessments of a patient’s health status or to inform treatment choices [87,88]. Future research should aim to converge on a smaller, purpose-driven subset of digital markers that demonstrably predict clinically relevant states. This principled narrowing is essential both to protect patient privacy and to ensure that only the most informative digital markers enter routine clinical practice.

### Operationalizing Digital Markers for Clinical Use

Moving beyond passive monitoring and toward meaningful clinical use requires digital markers to become actionable tools that can inform treatment decisions. Passive signals must first be validated as reliable indicators of mood state changes across diverse and representative populations. Several studies in this review demonstrated the potential for mood state classification [38,55], but few linked their results to concrete clinical actions. To be actionable, there must be predefined interventions for each digital marker, such as clinician alerts or medication adjustments. There will need to be extensive collaboration between data scientists, clinicians, ethicists, and patients to establish clinically meaningful thresholds for action (eg, the threshold for deviation in HRV that warrants a clinical intervention). Real-time monitoring must be supported by an infrastructure that enables clinicians to integrate these interventions into care delivery systems in a minimally invasive way, balancing accuracy and avoiding overburdening of patients or clinicians [81]. Successful clinical integration will also require regulatory pathways that can establish safety and effectiveness, as digital markers can only improve outcomes if embedded within care models that translate detected mood state changes into clinical action. Overall, using digital markers for intervention will require technical precision and careful integration into existing health care systems.

### Limitations of This Review

We chose to exclude studies that focused on unipolar depression and/or entangled BD results with other disorders. While the exclusion allowed for a more focused review, it may have omitted relevant findings or generalizable methods that could inform BD-specific monitoring strategies. Additionally, because cyclothymia was rarely represented in the eligible literature, our conclusions primarily apply to BDI and BDII rather than the full bipolar spectrum. Other related reviews solely focus on monitoring depression [80], suicidality [89], or psychosis [90]. We chose to include some studies with methods that are not directly possible for remote monitoring (eg, external photometers and microphones) but could be made possible using alternative sensors (eg, via smartphone or wearable microphones). Additionally, we identified a high risk of bias in nearly all studies due to low sample and event sizes, especially studies that developed predictive models (see Table S2 in Multimedia Appendix 1). Hypomanic/manic episodes meeting diagnostic criteria may be less frequent than sub-threshold episodes and are therefore difficult to capture outside of the inpatient setting. However, the inpatient setting lacks ecological validity. Therefore, the digital markers presented in this review need to be further validated in larger and more diverse cohorts with longer and more frequent clinical touchpoints. Heterogeneity in patient follow-up, measurement protocols, and reporting of results may limit the reproducibility and comparison of findings across studies. For example, 8 different types of wearable devices are used in the included studies, as well as different types of smartphones (Android and Apple) and smartphone apps. Different devices often use proprietary algorithms to derive behavioral and physiological features, like sleep staging, leading to inconsistent outputs [91].

### Conclusion and Future Directions

Digital markers derived from smartphone and wearable sensors present a promising pathway toward precision psychiatry in BD, enabling continuous, real-time monitoring of mood states. This review highlights several categories of digital markers that appear clinically promising, such as physical activity, sleep, and speech. In particular, markers tied to circadian rhythms (nighttime light exposure and sleep-wake cycles) have demonstrated robust predictive power across studies and align with diagnostic criteria. Researchers and clinicians need to establish which objective digital markers have clinical significance to improve psychiatric practice through longitudinal clinical trials in larger cohorts. Next steps should include standardizing features across devices, validating models across diverse populations, and integrating passive monitoring into clinical workflows. Ethical implementation will also require balancing privacy with clinical actionability. ML and AI models for predicting mood states are promising but need to be further validated, and can be leveraged in the future to implement the digital markers summarized in this review for improved performance. Remote monitoring has the potential to fundamentally shift the clinical management of BD by enabling earlier detection of mood state transitions and real-time risk assessment, revolutionizing how psychiatric care is delivered.

## Supplementary material

10.2196/94819 Multimedia Appendix 1 Search strategy, study characteristics, and risk-of-bias assessments.

10.2196/94819 Checklist 1 PRISMA checklist.

## Abbreviations

APPLE: Association between the Pathology of Bipolar Disorder and Light Exposure in Daily Life
ASRM: Altman Self-Rating Mania Scale
AUROC: area under the receiver operating characteristic curve
BD: bipolar disorder
BDI: bipolar I disorder
BDII: bipolar II disorder
BiMM: binary mixed model
BPD: borderline personality disorder
BRMS: Bech-Rafaelsen Mania Scale
DSM-5: *Diagnostic and Statistical Manual of Mental Disorders* (Fifth Edition)
EDA: electrodermal activity
HC: healthy controls
HDRS: Hamilton Depression Rating Scale
HR: heart rate
HRV: heart rate variability
IDS-C: Inventory of Depressive Symptomatology–Clinician Rated
MADRS: Montgomery-Åsberg Depression Rating Scale
MDCRC: Mood Disorder Cohort Research Consortium
MDD: major depressive disorder
ML: machine learning
NOS: Newcastle-Ottawa Scale
OCD: obsessive-compulsive disorder
PHQ-8: Patient Health Questionnaire-8
PHQ-9: Patient Health Questionnaire-9
PRISMA: Preferred Reporting Items for Systematic Reviews and Meta-Analyses
PROBAST: Prediction Model Risk of Bias Assessment Tool
PROSPERO: International Prospective Register of Systematic Reviews
QIDS: Quick Inventory of Depressive Symptomatology
RADAR: Remote Assessment of Disease and Relapse
REM: rapid eye movement
SCID: Structured Clinical Interview for *DSM* Disorders
XAI: explainable AI
XGBoost: extreme gradient boosting
YMRS: Young Mania Rating Scale

## References

[1] Grande I, Berk M, Birmaher B, Vieta E (2016). Bipolar disorder. The Lancet.

[2] Vieta E, Berk M, Schulze TG (2018). Bipolar disorders. Nat Rev Dis Primers.

[3] Judd LL, Akiskal HS, Schettler PJ (2002). The long-term natural history of the weekly symptomatic status of bipolar I disorder. Arch Gen Psychiatry.

[4] Judd LL, Akiskal HS, Schettler PJ (2003). A prospective investigation of the natural history of the long-term weekly symptomatic status of bipolar II disorder. Arch Gen Psychiatry.

[5] Simon GE, Hunkeler E, Fireman B, Lee JY, Savarino J (2007). Risk of suicide attempt and suicide death in patients treated for bipolar disorder. Bipolar Disord.

[6] Plans L, Barrot C, Nieto E (2019). Association between completed suicide and bipolar disorder: a systematic review of the literature. J Affect Disord.

[7] Ortiz A, Mulsant BH (2024). Beyond step count: are we ready to use digital phenotyping to make actionable individual predictions in psychiatry?. J Med Internet Res.

[8] Bauer M, Andreassen OA, Geddes JR (2018). Areas of uncertainties and unmet needs in bipolar disorders: clinical and research perspectives. Lancet Psychiatry.

[9] Faurholt-Jepsen M, Frost M, Ritz C (2015). Daily electronic self-monitoring in bipolar disorder using smartphones - the MONARCA I trial: a randomized, placebo-controlled, single-blind, parallel group trial. Psychol Med.

[10] Faurholt-Jepsen M, Frost M, Christensen EM, Bardram JE, Vinberg M, Kessing LV (2020). The effect of smartphone-based monitoring on illness activity in bipolar disorder: the MONARCA II randomized controlled single-blinded trial. Psychol Med.

[11] Peralta V, Cuesta MJ (1998). Lack of insight in mood disorders. J Affect Disord.

[12] Saccaro LF, Amatori G, Cappelli A, Mazziotti R, Dell’Osso L, Rutigliano G (2021). Portable technologies for digital phenotyping of bipolar disorder: a systematic review. J Affect Disord.

[13] Ortiz A, Maslej MM, Husain MI, Daskalakis ZJ, Mulsant BH (2021). Apps and gaps in bipolar disorder: a systematic review on electronic monitoring for episode prediction. J Affect Disord.

[14] Page MJ, McKenzie JE, Bossuyt PM (2021). The PRISMA 2020 statement: an updated guideline for reporting systematic reviews. BMJ.

[15] Ouzzani M, Hammady H, Fedorowicz Z, Elmagarmid A (2016). Rayyan-a web and mobile app for systematic reviews. Syst Rev.

[16] Moons KGM, Wolff RF, Riley RD (2019). PROBAST: a tool to assess risk of bias and applicability of prediction model studies: explanation and elaboration. Ann Intern Med.

[17] Wells G, Shea B, O’Connel D (2000). The newcastle-ottawa scale (NOS) for assessing the quality of nonrandomised studies in meta-analyses. Ottawa Hospital Research Institute (OHRI).

[18] Valenza G, Nardelli M, Lanatà A (2014). Wearable monitoring for mood recognition in bipolar disorder based on history-dependent long-term heart rate variability analysis. IEEE J Biomed Health Inform.

[19] Anmella G, Mas A, Sanabra M (2024). Electrodermal activity in bipolar disorder: Differences between mood episodes and clinical remission using a wearable device in a real-world clinical setting. J Affect Disord.

[20] Anmella G, Corponi F, Li BM (2023). Exploring digital biomarkers of illness activity in mood episodes: hypotheses generating and model development study. JMIR Mhealth Uhealth.

[21] Côté-Allard U, Jakobsen P, Stautland A (2022). Long–short ensemble network for bipolar manic-euthymic state recognition based on wrist-worn sensors. IEEE Pervasive Comput.

[22] Faurholt-Jepsen M, Frost M, Vinberg M, Christensen EM, Bardram JE, Kessing LV (2014). Smartphone data as objective measures of bipolar disorder symptoms. Psychiatry Res.

[23] Faurholt-Jepsen M, Vinberg M, Frost M (2016). Behavioral activities collected through smartphones and the association with illness activity in bipolar disorder. Int J Methods Psychiatr Res.

[24] Faurholt-Jepsen M, Busk J, Vinberg M (2021). Daily mobility patterns in patients with bipolar disorder and healthy individuals. J Affect Disord.

[25] Palmius N, Tsanas A, Saunders KEA (2017). Detecting bipolar depression from geographic location data. IEEE Trans Biomed Eng.

[26] Beiwinkel T, Kindermann S, Maier A (2016). Using smartphones to monitor bipolar disorder symptoms: a pilot study. JMIR Ment Health.

[27] Ludwig VM, Bittendorf CA, Reinhard I (2025). Early warning signals of bipolar relapse: investigating critical slowing down in smartphone data. J Affect Disord.

[28] Ludwig VM, Bittendorf CA, Reinhard I (2025). Predicting depressive and manic episodes in patients with bipolar disorder using statistical process control methods on passive sensing data. J Psychopathol Clin Sci.

[29] Aledavood T, Luong N, Baryshnikov I (2025). Multimodal digital phenotyping study in patients with major depressive episodes and healthy controls (mobile monitoring of mood): observational longitudinal study. JMIR Ment Health.

[30] Faurholt-Jepsen M, Busk J, Rohani DA (2022). Differences in mobility patterns according to machine learning models in patients with bipolar disorder and patients with unipolar disorder. J Affect Disord.

[31] Lee TY, Chen CH, Liu CM (2025). Fourier transform analysis of GPS-derived mobility patterns for diagnosis and mood monitoring of bipolar and major depressive disorders: prospective study. J Med Internet Res.

[32] Faurholt-Jepsen M, Brage S, Kessing LV, Munkholm K (2017). State-related differences in heart rate variability in bipolar disorder. J Psychiatr Res.

[33] Corponi F, Li BM, Anmella G (2024). A Bayesian analysis of heart rate variability changes over acute episodes of bipolar disorder. Npj Ment Health Res.

[34] Valenza G, Nardelli M, Lanata’ A (2016). Predicting mood changes in bipolar disorder through heartbeat nonlinear dynamics. IEEE J Biomed Health Inform.

[35] Nardelli M, Lanata A, Bertschy G, Scilingo EP, Valenza G (2017). Heartbeat complexity modulation in bipolar disorder during daytime and nighttime. Sci Rep.

[36] Cho CH, Lee T, Kim MG, In HP, Kim L, Lee HJ (2019). Mood prediction of patients with mood disorders by machine learning using passive digital phenotypes based on the circadian rhythm: prospective observational cohort study. J Med Internet Res.

[37] Lee HJ, Cho CH, Lee T (2023). Prediction of impending mood episode recurrence using real-time digital phenotypes in major depression and bipolar disorders in South Korea: a prospective nationwide cohort study. Psychol Med.

[38] Lipschitz JM, Lin S, Saghafian S, Pike CK, Burdick KE (2025). Digital phenotyping in bipolar disorder: using longitudinal Fitbit data and personalized machine learning to predict mood symptomatology. Acta Psychiatr Scand.

[39] Esaki Y, Obayashi K, Saeki K, Fujita K, Iwata N, Kitajima T (2020). Association between light exposure at night and manic symptoms in bipolar disorder: cross-sectional analysis of the APPLE cohort. Chronobiol Int.

[40] Esaki Y, Obayashi K, Saeki K, Fujita K, Iwata N, Kitajima T (2022). Effect of nighttime bedroom light exposure on mood episode relapses in bipolar disorder. Acta Psychiatr Scand.

[41] Esaki Y, Kitajima T, Obayashi K, Saeki K, Fujita K, Iwata N (2019). Daytime light exposure in daily life and depressive symptoms in bipolar disorder: a cross-sectional analysis in the APPLE cohort. J Psychiatr Res.

[42] Esaki Y, Obayashi K, Saeki K, Fujita K, Iwata N, Kitajima T (2021). Preventive effect of morning light exposure on relapse into depressive episode in bipolar disorder. Acta Psychiatr Scand.

[43] Kim B, Chae M, Kim Y Early prediction of depressive episodes in mood disorders using circadian rhythm indicators and deep learning.

[44] Ebner-Priemer UW, Mühlbauer E, Neubauer AB (2020). Digital phenotyping: towards replicable findings with comprehensive assessments and integrative models in bipolar disorders. Int J Bipolar Disord.

[45] Faurholt-Jepsen M, Brage S, Vinberg M, Kessing LV (2016). State-related differences in the level of psychomotor activity in patients with bipolar disorder - continuous heart rate and movement monitoring. Psychiatry Res.

[46] Gershon A, Ram N, Johnson SL, Harvey AG, Zeitzer JM (2016). Daily actigraphy profiles distinguish depressive and interepisode states in bipolar disorder. Clin Psychol Sci.

[47] Clemens J, Mühlbauer E, Reinhard I (2025). Circadian rhythm parameters differentiate euthymic, manic and depressive mood states in bipolar disorders - an explorative pilot study. Int J Bipolar Disord.

[48] Jakobsen P, Stautland A, Riegler MA (2022). Complexity and variability analyses of motor activity distinguish mood states in bipolar disorder. PLoS One.

[49] Ortiz A, Halabi R, Alda M (2025). Day-to-day variability in sleep and activity predict the onset of a hypomanic episode in patients with bipolar disorder. J Affect Disord.

[50] Esaki Y, Obayashi K, Saeki K, Fujita K, Iwata N, Kitajima T (2021). Association between circadian activity rhythms and mood episode relapse in bipolar disorder: a 12-month prospective cohort study. Transl Psychiatry.

[51] Tanaka T, Kokubo K, Iwasa K, Sawa K, Yamada N, Komori M (2018). Intraday activity levels may better reflect the differences between major depressive disorder and bipolar disorder than average daily activity levels. Front Psychol.

[52] Jones SH, Hare DJ, Evershed K (2005). Actigraphic assessment of circadian activity and sleep patterns in bipolar disorder. Bipolar Disord.

[53] Esaki Y, Obayashi K, Saeki K, Fujita K, Iwata N, Kitajima T (2023). Circadian variability of objective sleep measures predicts the relapse of a mood episode in bipolar disorder: findings from the APPLE cohort. Psychiatry Clin Neurosci.

[54] Esaki Y, Obayashi K, Saeki K, Fujita K, Iwata N, Kitajima T (2024). Daytime napping and depressive symptoms in bipolar disorder: a cross-sectional analysis of the APPLE cohort. Sleep Med.

[55] Lim D, Jeong J, Song YM (2024). Accurately predicting mood episodes in mood disorder patients using wearable sleep and circadian rhythm features. NPJ Digit Med.

[56] Ikäheimonen A, Luong N, Baryshnikov I (2024). Predicting and monitoring symptoms in patients diagnosed with depression using smartphone data: observational study. J Med Internet Res.

[57] Liu Q, Ning E, Ross MK (2024). Digital phenotypes of mobile keyboard backspace rates and their associations with symptoms of mood disorder: algorithm development and validation. J Med Internet Res.

[58] Stange JP, Zulueta J, Langenecker SA (2018). Let your fingers do the talking: passive typing instability predicts future mood outcomes. Bipolar Disord.

[59] Zulueta J, Piscitello A, Rasic M (2018). Predicting mood disturbance severity with mobile phone keystroke metadata: a biaffect digital phenotyping study. J Med Internet Res.

[60] Cao B, Zheng L, Zhang C DeepMood: modeling mobile phone typing dynamics for mood detection.

[61] Faurholt‐Jepsen M, Vinberg M, Frost M, Christensen EM, Bardram JE, Kessing LV (2015). Smartphone data as an electronic biomarker of illness activity in bipolar disorder. Bipolar Disord.

[62] Dominiak M, Kaczmarek-Majer K, Antosik-Wójcińska AZ (2022). Behavioral and self-reported data collected from smartphones for the assessment of depressive and manic symptoms in patients with bipolar disorder: prospective observational study. J Med Internet Res.

[63] Grünerbl A, Muaremi A, Osmani V (2015). Smartphone-based recognition of states and state changes in bipolar disorder patients. IEEE J Biomed Health Inform.

[64] Faurholt-Jepsen M, Rohani DA, Busk J (2024). Using digital phenotyping to classify bipolar disorder and unipolar disorder – exploratory findings using machine learning models. Eur Neuropsychopharmacol.

[65] Zhang J, Pan Z, Gui C (2018). Analysis on speech signal features of manic patients. J Psychiatr Res.

[66] Kaczmarek-Majer K, Dominiak M, Antosik AZ (2025). Acoustic features from speech as markers of depressive and manic symptoms in bipolar disorder: a prospective study. Acta Psychiatr Scand.

[67] Ji J, Dong W, Li J (2024). Depressive and mania mood state detection through voice as a biomarker using machine learning. Front Neurol.

[68] Faurholt-Jepsen M, Busk J, Frost M (2016). Voice analysis as an objective state marker in bipolar disorder. Transl Psychiatry.

[69] Karam ZN, Provost EM, Singh S Ecologically valid long-term mood monitoring of individuals with bipolar disorder using speech.

[70] Pan Z, Gui C, Zhang J, Zhu J, Cui D (2018). Detecting manic state of bipolar disorder based on support vector machine and Gaussian mixture model using spontaneous speech. Psychiatry Investig.

[71] Faurholt-Jepsen M, Rohani DA, Busk J, Vinberg M, Bardram JE, Kessing LV (2021). Voice analyses using smartphone-based data in patients with bipolar disorder, unaffected relatives and healthy control individuals, and during different affective states. Int J Bipolar Disord.

[72] Faurholt-Jepsen M, Rohani DA, Busk J (2022). Discriminating between patients with unipolar disorder, bipolar disorder, and healthy control individuals based on voice features collected from naturalistic smartphone calls. Acta Psychiatr Scand.

[73] Wu CT, Hsieh MH, Chen IM (2025). Using wearable device and machine learning to predict mood symptoms in bipolar disorder: development and usability study. JMIR Med Inform.

[74] Langholm C, Breitinger S, Gray L (2023). Classifying and clustering mood disorder patients using smartphone data from a feasibility study. NPJ Digit Med.

[75] Cho CH, Ahn YM, Kim SJ (2017). Design and Methods of the Mood Disorder Cohort Research Consortium (MDCRC) study. Psychiatry Investig.

[76] (2013). Diagnostic and Statistical Manual of Mental Disorders.

[77] Takaesu Y (2018). Circadian rhythm in bipolar disorder: a review of the literature. Psychiatry Clin Neurosci.

[78] Thorell LH, Kjellman BF, d’Elia G (1987). Electrodermal activity in antidepressant medicated and unmedicated depressive patients and in matched healthy subjects. Acta Psychiatr Scand.

[79] Provenza NR, Sheth SA, Dastin-van Rijn EM (2021). Long-term ecological assessment of intracranial electrophysiology synchronized to behavioral markers in obsessive-compulsive disorder. Nat Med.

[80] Abd-Alrazaq A, AlSaad R, Shuweihdi F, Ahmed A, Aziz S, Sheikh J (2023). Systematic review and meta-analysis of performance of wearable artificial intelligence in detecting and predicting depression. NPJ Digit Med.

[81] Woll S, Birkenmaier D, Biri G (2025). Applying AI in the context of the association between device-based assessment of physical activity and mental health: systematic review. JMIR Mhealth Uhealth.

[82] Bartoli F, Cavaleri D, Crocamo C (2025). Artificial intelligence and bipolar disorder: applications of machine learning models for diagnosis, treatment, and outcome prediction. Alpha Psychiatry.

[83] Sempionatto JR, Lasalde-Ramírez JA, Mahato K, Wang J, Gao W (2022). Wearable chemical sensors for biomarker discovery in the omics era. Nat Rev Chem.

[84] Provenza NR, Reddy S, Allam AK (2024). Disruption of neural periodicity predicts clinical response after deep brain stimulation for obsessive-compulsive disorder. Nat Med.

[85] Matcham F, Barattieri di San Pietro C, Bulgari V (2019). Remote assessment of disease and relapse in major depressive disorder (RADAR-MDD): a multi-centre prospective cohort study protocol. BMC Psychiatry.

[86] Hurley ME, Sonig A, Herrington J (2024). Ethical considerations for integrating multimodal computer perception and neurotechnology. Front Hum Neurosci.

[87] Kostick-Quenet KM, Herrington J, Storch EA (2024). Personalized roadmaps for returning results from digital phenotyping. Am J Bioeth.

[88] Martinez-Martin N, Insel TR, Dagum P, Greely HT, Cho MK (2018). Data mining for health: staking out the ethical territory of digital phenotyping. NPJ Digital Med.

[89] Büscher R, Winkler T, Mocellin J (2024). A systematic review on passive sensing for the prediction of suicidal thoughts and behaviors. NPJ Mental Health Res.

[90] Bladon S, Eisner E, Bucci S (2025). A systematic review of passive data for remote monitoring in psychosis and schizophrenia. NPJ Digit Med.

[91] Schyvens AM, Peters B, Van Oost NC (2025). A performance validation of six commercial wrist-worn wearable sleep-tracking devices for sleep stage scoring compared to polysomnography. Sleep Adv.

