# Supplementary material for: Digital Markers for Passive Remote Monitoring of Bipolar Disorder: Systematic Review
Source: JMIR Ment Health. 2026 Aug 13;13:e94819. doi: 10.2196/94819 (PMC13472530; doi:10.2196/94819)
Supplement: Supplementary file 1 [file mental-v13-e94819-s001.docx]

Search terms

**Field restriction.** All terms are limited to the **Title/Abstract** field to prioritize topical relevance and reduce noise from full-text indexing. MeSH limits are used only for Humans and Adults.

**Concept blocks (combined as C1 AND C2 AND C3):**

- **C1: Bipolar disorder diagnosis terms.**
  Captures BD regardless of subtype:
  "bipolar disorder" [Title/Abstract] OR "bipolar depression" [Title/Abstract] OR "bipolar I disorder" [Title/Abstract] OR "bipolar II disorder" [Title/Abstract] OR "bipolar 1 disorder" [Title/Abstract] OR "bipolar 2 disorder" [Title/Abstract] OR mania [Title/Abstract]
- **C2: Behavioral/physiological signals.**
  Encompasses the feature domains used in remote/passive monitoring (mood/affect, sleep/circadian, activity/motor/mobility, socialization, speech/voice, vision/face, physiology incl. HR/HRV/EDA, light exposure, device usage):
  (mood [Title/Abstract] OR emotion [Title/Abstract] OR physiology [Title/Abstract] OR "heart rate" [Title/Abstract] OR electrophysiology [Title/Abstract] OR electrophysiological [Title/Abstract] OR behavior [Title/Abstract] OR activity [Title/Abstract] OR motor [Title/Abstract] OR sleep [Title/Abstract] OR energy [Title/Abstract] OR socialization [Title/Abstract] OR social [Title/Abstract] OR speech [Title/Abstract] OR movement [Title/Abstract] OR face [Title/Abstract] OR "facial expression" [Title/Abstract] OR location [Title/Abstract] OR geolocation [Title/Abstract] OR function [Title/Abstract] OR HRV [Title/Abstract] OR wellbeing [Title/Abstract] OR "device usage" [Title/Abstract] OR light [Title/Abstract])
- **C3: Monitoring/assessment intent.**
  Focuses results on measurement and biomarker aims rather than unrelated mentions:
  (tracking [Title/Abstract] OR monitoring [Title/Abstract] OR assessment [Title/Abstract] OR observation [Title/Abstract] OR detection [Title/Abstract] OR identification [Title/Abstract] OR marker [Title/Abstract] OR measuring [Title/Abstract])

**Filters.** Publication year, language, species, and age group were restricted as follows:

- AND ("2000" [Date - Publication] : "3000" [Date - Publication]) AND (English [Language]) AND (Humans [MeSH Terms]) AND (Adult [MeSH Terms])

PubMed example search:

**Search terms**

("bipolar disorder" [Title/Abstract] OR "bipolar depression" [Title/Abstract] OR "bipolar I disorder" [Title/Abstract] OR "bipolar II disorder" [Title/Abstract] OR "bipolar 1 disorder" [Title/Abstract] OR "bipolar 2 disorder" [Title/Abstract] OR mania [Title/Abstract])

AND (mood [Title/Abstract] OR emotion [Title/Abstract] OR physiology [Title/Abstract] OR "heart rate" [Title/Abstract] OR electrophysiology [Title/Abstract] OR electrophysiological [Title/Abstract] OR behavior [Title/Abstract] OR activity [Title/Abstract] OR motor [Title/Abstract] OR sleep [Title/Abstract] OR energy [Title/Abstract] OR socialization [Title/Abstract] OR social [Title/Abstract] OR speech [Title/Abstract] OR movement [Title/Abstract] OR face [Title/Abstract] OR "facial expression" [Title/Abstract] OR location [Title/Abstract] OR geolocation [Title/Abstract] OR function [Title/Abstract] OR HRV [Title/Abstract] OR wellbeing [Title/Abstract] OR “device usage” [Title/Abstract] OR light)

AND (tracking [Title/Abstract] OR monitoring [Title/Abstract] OR assessment [Title/Abstract] OR observation [Title/Abstract] OR detection [Title/Abstract] OR identification [Title/Abstract] OR marker [Title/Abstract] OR measuring [Title/Abstract])

AND ("2000" [Date - Publication] : "3000" [Date - Publication]) AND (English [Language]) AND (Humans [MeSH Terms]) AND (Adult [MeSH Terms])

**Table S1:** Included Study Characteristics

| Author (Year) | Marker Category: Description | Sensing Methods | Statistical/ML Methods | Clinical Scales (Frequency) | Sample |
| --- | --- | --- | --- | --- | --- |
| Aledavood et al (2025) [29] | **Multi-Marker (Geolocation, Smartphone Usage, Physical Activity):** Weekday location variance and normalized location entropy were lower in patients vs controls (location variance: patient mean −11.91 vs control −10.04, P=.004; normalized entropy: patient 1.57 vs control 2.10, P=.05). Morning accelerometer magnitude variability (SD of phone accelerometer magnitude) was negatively associated with PHQ-9 (β=−2.05, 95% CI −4.18 to −0.20; P=.02). Group-level smartphone use volume (screen time, app categories) did not significantly differ, but temporal “rhythms” differed (patients had less consistent screen-use rhythms; controls showed different weekly app-use patterns). Depression severity was associated with call behavior in the preceding 14 days: incoming call duration was negatively associated (β=−0.08, 95% CI −0.12 to −0.04; P<.001) and outgoing call duration was positively associated (β=0.05, 95% CI 0.00 to 0.09; P=.02). | *Device:* Smartphone (Android)  *Measurements:* screen events, apps, calls/SMS metadata, GPS/location, accelerometer, battery  *Data Characteristics:* collected for up to 365 days  *Missing Data:* average of 10.5% across all patients, 4.5% for BD | Linear mixed effects model, Mann-Whitney U tests | PHQ-9 (biweekly) | N = 151 (21 BD, 76 MDD, 24 BPD, 30 HC) |
| Anmella et al (2023) [20] | **Multi-Marker Classification (Physical Activity, Electrodermal Activity, Heart Rate):** BiLSTM trained on wearable data at 3 acute timepoints acheived 61-70% accuracy at classifying severity of an acute affective episode. Most important features were accelleration, electrodermal activity, and HR. | *Device*: Wearable (Empatica E4)  *Measurements*: Acceleration, skin temperature, blood volume pulse, heart rate, and electrodermal activity  *Data Characteristics*: 48hr recordings at acute episode, response, and remission  *Missing Data:* median of 11.05% of data lost due to quality control | Bidirectional Long-Short Term Memory neural network | YMRS, HDRS (at 3 timepoints) | N = 8, inpatients |
| Anmella et al (2024) [19] | **Electrodermal Activity:** Patients with bipolar depression showed significantly reduced mean EDA and EDA peaks per minute, which increased to levels similar to euthymia or healthy controls after clinical remission | *Device*: Wearable (Empatica E4)  *Measurements*: Acceleration, skin temperature, blood volume pulse, heart rate, and electrodermal activity  *Data Characteristics*: 48hr recordings at acute episode and remission  *Missing Data:* median percentage of data per recording session dropped from further analysis due to artifact detection was 31.89 | ANOVA, paired t-tests | YMRS, HDRS (at 2 timepoints) | N = 49, inpatients |
| Beiwinkel et al (2016) [26] | **Multi-Marker (Geolocation, Smartphone Usage):** Smartphone-based measures (self-reported mood, social communication, and physical activity) were closely linked to both depressive and manic symptoms. Specifically, lower mood, fewer outgoing texts, and reduced movement predicted higher or increasing depressive symptoms, while lower physical activity but higher social communication predicted higher or increasing manic symptoms. | *Device:* Smartphone (Social Information Monitoring for Patients with Bipolar Affective Disorder app)  *Measurements:* movement activity: GPS, cell tower movement, accelerometry, and socialization: number and duration of outgoing calls and the number of SMS sent per day  *Data Characteristics:* Daily data was collected for up to 12 months  *Missing Data:* 55.7% compliance rate | Random-coefficient multilevel models | YMRS, HAMD (every 8 weeks) | N = 13, outpatients |
| Cao et al (2017) [60] | **Smartphone Usage:** This study uses a deep neural network trained on smartphone keyboard typing features to classify mild/severe depression (90% accuracy) and predict YMRS scores. | Device: Smartphone (BiAffect app on Android smartphones)  Measurements: keyboard strokes passively collected  *Data Characteristics:* passively collected over 8 weeks  *Missing Data:* not stated | A novel "DeepMood' deep learning architecture, utilizing Gated Recurrent Units (GRU) | HDRS, YMRS (weekly) | N = 12 (7 BD I, 5 BD II), outpatient |
| Cho et al (2019) [36] | **Multi-Marker Classification (Physical Activity, Sleep, Heart Rate, Light Exposure):** Features related to circadian rhythms were able to classify mood episodes in BDII and BDII with moderate accuracy. | *Device:* Wearable (Fitbit Charge), Smartphone (Android)  *Measurements:* 4 different categories related to circadian rhythms: light exposure, steps, sleep, and heart rate  *Data Characteristics:* 2 years of daily watch measurements  *Missing Data:* went from 17,542 to 2,003 sample days after removing all days with any missing data | Random forest | YMRS, QIDS (every 12 weeks) | N = 37, outpatients |
| Clemens et al (2025) [47] | **Physical Activity:** Circadian rhythm parameters from wrist accelerometry differentiated daily mood states. Depressive days vs euthymia were associated with lower MeanDiff (reduced overall activity; B = −0.02, P < .001), lower interdaily stability (IS) (B = −0.80, P = .009), and higher FormDiff (more rigid circadian form; B = 0.03, P < .001). (Hypo)manic days vs euthymia were associated with higher MeanDiff (B = 0.02, P = .007). | *Device:* Wearable (Movisens Move 3)  *Measurements:* accelerometry  *Data Characteristics:* continuously up to 1 year  *Missing Data:* went from 4,055 to 2,669 days of data after excluding days with >360 min non-wear time | Mixed-effects logistic models | YMRS, MADRS (biweekly) | N = 23, outpatients |
| Corponi et al (2024) [33] | **Heart Rate:** This paper establishes positive change in natural logarithm of the Root Mean Square of Successive RR interval Differences as a marker of symptom resolution regardless of episode polarity (95.175% probability of positive direction). | *Device*: Wearable (Empatica E4)  *Measurements*: Heart Rate Variability  *Data Characteristics*: 48 hour recordings at 4 acute timepoints  *Missing Data:* median percentage of data excluded due to quality control was 9.05% | Bayesian model | YMRS, HDRS (up to four times: acute phase, clinical response, remission, euthymia) | N = 23, inpatients |
| Cote-Allard et al (2022) [21] | **Multi-Marker Classification (Physical Activity, Electrodermal Activity, Heart Rate):** This work establishes a deep learning methodology that used wearable data to distinguish manic and euthymic states across (not within) patients. | *Device:* Wearable (Empatica E4)  *Measurements*: Accelerometry, Electrodermal Activity, PPG, HR  *Data Characteristics*: 24 hour recordings  *Missing Data:* n/a | Long–Short Term Memory | YMRS, MADRS (one time point) | N = 47, inpatients |
| Dominiak et al (2022) [62] | **Smartphone Usage:** Smartphone socialization data collected in the 7 days before and 2 days after a clinical visit shows significant differences in depressive, euthymic, and mixed/manic states. | *Device:* Smartphone (BDmon app)  *Measurements*: Calls and text message statistics, including incoming answered and missed calls, and outgoing calls and text messages  *Data Characteristics*: Data was collected for the 7 days before and 2 days after a clinical evaluation, with a minimum of one evaluation per participant  *Missing Data:* varying degrees of completeness across markers | Linear mixed-effects model | YMRS, HDRS (every 3 months, or after a mood change was suspected after a fortnightly phone call intervention) | N = 51, outpatients |
| Ebner-Priemer et al (2020) [44] | **Physical Activity:** Passively tracked activity metrics were statistically meaningfully associated with same-day manic and depressive psychopathology. | *Device:* Smartphone (movisensXS app)  *Measurements:* Incoming and outgoing phone calls and text messages, number of different call and text contacts, frequency and duration of times the display was on/off, rates of transmitted and received data, travel distances in kilometers, frequency and duration of different activity classes (in vehicle, on bicycle, walking, still, unknown, tilting) and the velocity of movement and number of steps  *Data Characteristics:* 12 month monitoring period, daily recordings  *Missing Data:* 99% compliance for smartphone data | Multilevel structural equation monitoring | YMRS, BRMRS, MADRS (every 2 weeks) | N = 29, outpatients |
| Esaki et al (2019) [41] | **Light Exposure:** Higher daytime light intensity is associated with lower depression severity across (not within) patients. Depression only. | *Device:* Wearable (Actiwatch Spectrum Plus)  *Measurements:* light exposure  *Data Characteristics:* 7 consecutive days  *Missing Data:* n/a | t-test, Mann-Whitney U test | MADRS (one time point) | N = 181, inpatients |
| Esaki et al (2020) [39] | **Light Exposure:** Bedroom light exposure at night is significantly associated with manic symptoms. Mania only. | *Device:* Portable Photometer (LX-28SD)  *Measurements:* light exposure  *Data Characteristics:* 7 consecutive days  *Missing Data: n/a* | t-test, Mann-Whitney U test | YMRS (one time point) | N = 184, outpatients |
| Esaki et al (2021a) [50] | **Physical Activity:** A later timing of circadian activity rhythm (M10 onset time) was significantly associated with future depressive episode relapses. | *Device:* Wearable (Actiwatch Spectrum Plus)  *Measurements:* Accelerometry  *Data Characteristics:* 7 consecutive days at baseline and follow-up  *Missing Data: n/a* | Cox proportional hazards model | YMRS, MADRS (at baseline and at 12 month follow-up) | N = 189, outpatients |
| Esaki et al (2021b) [42] | **Light Exposure:** Higher daytime light intensity is associated with lower likelihood of depression relapse across (not within) patients. Depression only. | *Device:* Wearable (Actiwatch Spectrum Plus)  *Measurements:* light exposure  *Data Characteristics:* 7 consecutive days  *Missing Data: n/a* | Cox proportional hazards model | MADRS (at baseline and at 12 month follow-up) | N = 198, outpatients |
| Esaki et al (2022) [40] | **Light Exposure:** Bedroom light exposure at night is significantly associated with future manic episodes. Mania only. | *Device:* Portable Photometer (LX-28SD)  *Measurements:* light exposure  *Data Characteristics:* 7 consecutive days at baseline and follow-up  *Missing Data: n/a* | Cox proportional hazards model | YMRS (at baseline and at 2 year follow-up) | N = 157, outpatients |
| Esaki et al (2023) [53] | **Sleep:** Variability in total sleep time was significantly associated with an increase in the mood episode relapses, and participants with higher variability in total sleep time had a significantly shorter mean estimated time to mood episode relapse. | *Device:* Wearable (Actiwatch Spectrum Plus)  *Measurements:* Accelerometry  *Data Characteristics:* 7 consecutive days at baseline and follow-up  *Missing Data: n/a* | Cox proportional hazards model | YMRS, MADRS (at baseline and at 2 year follow-up) | N = 193, outpatients |
| Esaki et al (2024) [54] | **Sleep:** There is a significant association between daytime napping and depressive symptoms in patients with bipolar disorder. Specifically, in a multivariable logistic regression analysis, as the number of nap days, number of naps per day, and nap duration increased, the odds ratio (OR) for depressed state significantly increased. | *Device:* Wearable (Actiwatch Spectrum Plus)  *Measurements:* Accelerometry  *Data Characteristics:* 7 consecutive days at baseline  *Missing Data: n/a* | Cox proportional hazards model | YMRS, MADRS (at baseline) | N = 204, outpatients |
| Faurholt-Jepsen et al (2014) [22] | **Geolocation:** Change in smartphone cell tower ID is negatively correlated with depression severity. | *Device:* Smartphone (MONARCA app)  *Measurements:* Geolocation  *Data Characteristics:* Daily for 3 months  *Missing Data: n/a* | Mixed effect regression models | YMRS, HDRS (every 2 weeks) | N = 17, outpatients |
| Faurholt-Jepsen et al (2015) [61] | **Smartphone Usage:** There are significant correlations between smartphone socialization metrics and depressive and manic symptoms. | *Device:* Smartphone (MONARCA app)  *Measurements:* number of incoming and outgoing phone calls per day, the duration of incoming and outgoing phone calls per day, and the number of outgoing text messages per day  *Data Characteristics:* Daily for 6 months  *Missing Data: n/a* | Mixed effect regression models | YMRS, HDRS (every 2 weeks) | N = 61, outpatients |
| Faurholt-Jepsen et al (2016b) [68] | **Speech:** Affective states were classified using voice features extracted during everyday life phone calls. Voice features were found to be more accurate, sensitive and specific in the classification of manic or mixed states (AUC = 0.89) compared to depressive states (AUC = 0.78). | *Device:* Smartphone (MONARCA app, openSMILE toolkit)  *Measurements:* voice features extracted from natural phone calls  *Data Characteristics:* Daily for 12 weeks  *Missing Data:* Of the 28 patients, 13 patients provided enough voice feature data to train at least one model for classification of affective states. | Random forest | YMRS, HDRS (every 2 weeks) | N = 28, outpatients |
| Faurholt-Jepsen et al (2016c) [45] | **Physical Activity:** There was a significant correlation between the severity of manic symptoms and energy expenditure and trunk acceleration, respectively (across patients). | *Device:* Wearable (Actiheart, mounted on thorax)  *Measurements:* Psychomotor activity (movement/acceleration (m/s2) and heart rate (bpm)  *Data Characteristics:* Minimum 3 continuous days during affective state  *Missing Data: n/a* | Mixed effect regression models | YMRS, HDRS (one time point) | N = 19, outpatients |
| Faurholt-Jepsen et al (2016a) [23] | **Multi-Marker (Smartphone Usage, Geolocation):** The more severe the depressive symptoms (1) the longer the smartphone’s screen was “on”/day, (2) more received incoming calls/day, (3) fewer outgoing calls/day were made, (4) less answered incoming calls/day, (5) the patients moved less between cell towers IDs/day. Conversely, the more severe the manic symptoms (1) more outgoing text messages/day sent, (2) the phone calls/day were longer, (3) the fewer number of characters in incoming text messages/day, (4) the lower duration of outgoing calls/day, (5) the patients moved more between cell towers IDs/day. | *Device:* Smartphone (MONARCA app)  *Measurements:* the number of incoming text messages/day; the number of outgoing text messages/day; the duration of phone calls/day (collected with a maximum of 120 seconds/call); the amount of time the smartphone’s screen was “on”/day; the number of times the smartphone’s screen was turned “on”/day; and the number of cell tower ID changes/day  *Data Characteristics:* Daily for 12 weeks  *Missing Data: n/a* | Mixed effect regression models | YMRS, HDRS (every 2 weeks) | N = 29, outpatients |
| Faurholt-Jepsen et al (2017) [32] | **Heart Rate:** HRV is significantly higher in a manic state relative to depressive and euthymic states. | *Device:* Wearable (Actiheart)  *Measurements:* HRV  *Data Characteristics:* Minimum 3 continuous days during affective state  *Missing Data: n/a* | Mixed effect regression models | YMRS, HDRS (one time point) | N = 16, outpatients |
| Faurholt-Jepsen et al (2021a) [24] | **Geolocation:** Patients with BD during a depressive state were less mobile compared with a euthymic state. Patients with BD during an affective state had lower location entropy compared with a euthymic state | *Device:* Smartphone (MONARCA app)  *Measurements:* location data via GPS, WiFi, cell tower  *Data Characteristics:* mean of 101 days per patient  *Missing Data: n/a* | Mixed effect regression models | YMRS, HDRS (one time point) | N = 48, outpatients |
| Faurholt-Jepsen et al (2021b) [71] | **Speech**: Voice features extracted from naturalistic smartphone phone calls were used to classify bipolar disorder vs healthy controls/unaffected relatives and to classify within-BD affective states (mania vs euthymia, depression vs euthymia). User-dependent models outperformed user-independent models; within BD, mania and depression vs euthymia were classified with modest AUCs (~0.66). | *Device:* Smartphone (Android)  *Measurements:* 6552 speech features from naturalistic phone calls including pitch, loudness, etc.  *Data Characteristics:* mean 157 days with at least one voice recording; range 1-972 days  Missing Data: n/a | Random Forest | HDRS-17, YMRS (at baseline) | N = 180 (121 BD, 38 HC, 21 unaffected relatives) |
| Faurholt-Jepsen et al (2022a) [30] | **Geolocation:** BD patients during a depressive state showed significantly lower mobility (p=0.027) | *Device:* Smartphone (Android)  *Measurements:* GPS, cell tower signals  *Data Characteristics:* raw location aggregated into daily patterns  *Missing Data:* n/a | Mixed effect regression models, Balanced bagging classifier | HDRS-17 (at baseline) | N = 140 (65 BD, 75 UD), outpatient |
| Faurholt-Jepsen et al (2022b) [72] | **Speech:** UD was classified from BD (specificity = 0.84, AUC = 0.58) using acoustic features. Found that personalized user-dependent models outperformed general ones | *Device:* Smartphone (Android)  *Measurements:* acoustic features  *Data Characteristics:* collected during naturalistic calls  Missing Data: n/a | Random Forest | HDRS-17, YMRS (at baseline), | N = 207 (48 UD, 121 BD, 38 HC), outpatient |
| Faurholt-Jepsen et al (2024) [64] | **Smartphone Usage:** BD patients in depressive states had significantly fewer outgoing phone calls/day compared to UD. In a depressive state, BD was classified with AUC 0.84 and during an euthymic state, they were classified with AUC of 0.87 | *Device:* Smartphone (Android)  *Measurements:* Call logs, SMS counts, Screen state (on/off)  *Data Characteristics:* collected for 6 months  Missing Data: n/a | Random Forest, Linear mixed effect regression | HDRS-17, YMRS (at baseline), | N = 138 (64 BD, 74 UD), outpatients |
| Gershon et al (2016) [46] | **Physical Activity:** Depressive days are distinguished from other mood states by an overall lower activity level, and a pattern of later activity onset, a midday elevation of activity, and low evening activity. No distinct within-person activity patterns were found for hypomanic/manic days. | *Device:* Wearable (Actiwatch)  *Measurements:* accelerometry  *Data Characteristics:* 2 consecutive months  Missing Data: 13% of the total days had >60 minutes missing data and were not analyzed | Functional principal components analysis | YMRS, IDS-C (every month) | N = 37, outpatients |
| Grünerbl et al (2015) [63] | **Multi-Marker Classification (Smartphone Usage, Geolocation, Physical Activity, Speech)**: The aim of the study was to identify recognition of states and changes. Using socialization  features, the authors get prediction accuracies of 76% by fusing all sensor modalities and state change detection precision and recall of over 97%. | *Device:* Smartphone (Android smartphone running custom logging application)  *Measurements:* number of phone calls, total length, average length, number of unique numbers  *Data Characteristics:* 12 weeks continously, over 800 days total  *Missing Data:* missing 13-65 days of data out of 84 per patient | Naive Bayes classifier | HAMD, YMRS (every 3 weeks) | N = 10, outpatient |
| Ikäheimonen et al (2024) [56] | **Multi-Marker Classification (Physical Activity, Geolocation, Smartphone Usage)**: XGBoost with feature selection was able to to classify depressed states across disorders (MDD, BPD, BD) with an F1 score of 0.82, with an overall F1 score of 0.67 for detecting state transitions (4 states: remains/becomes depressed/non-depressed). | *Device:* Smartphone (Android)  *Measurements:* accelerometry, app usage, battery levels, call log, geolocation, screen time  *Data Characteristics:* continuously up to 1 year  *Missing Data: "*PHQ-9 survey answer compliance dropped below 70% after 6 weeks (3 biweekly periods) had passed, and after that, it continued to decline steadily. Passive data collection compliance shows a pattern similar to answering the PHQ-9 survey. Most of the missing data occurred due to the participant dropping out of the study, while some participants had gaps in data collection. Notably, only a few participants remained in the study for the entire year. Also, the data collection for participants was incomplete due to missing features." | XGBoost | PHQ-9 (biweekly) | N = 133, (21 BD, 85 MDD, 27 MDD & BPD), outpatient |
| Jakobsen et al (2022) [48] | **Physical Activity:** Motor activity of mania is characterized by altered complexity and variability when compared within-subject to euthymia. | *Device:* Wearable (Empatica E4)  *Measurements:* accelerometry  *Data Characteristics:* 24 hour recordings at admission and remission  *Missing Data: n/a* | Paired-Samples T-tests, Wilcoxon Signed Rank Test | YMRS, MADRS (2 times) | N = 16, inpatients |
| Ji et al (2024) [67] | **Speech:** Detected depressive and manic states from real-world journal voice recordings with an overall accuracy of 86.1% for detecting depression and 90.3% for mania. | *Device:* Smartphone (MoodMirror app)  *Measurements:* audio recordings  *Data Characteristics:* prompted once a day  *Missing Data: n/a* | Chinese-speech-pretrained Gate Recurrent Unit model | QIDS, YMRS (on app daily) | N = 93, inpatient |
| Jones et al (2005) [52] | **Physical Activity:** BD patients were found to have less stable and more variable circadian activity patterns than controls. Regression analysis indicated that variability alone was a significant independent predictor of diagnostic group. There was evidence from raw activity data that bipolar patients were also less active than controls. These differences were not associated with levels of subsyndromal symptoms. Bipolar patients did not differ from controls on any of the sleep indices used. | *Device:* Wearable (Actiwatch)  *Measurements:* 15 second epochs of actigraphy data  *Data Characteristics:* 7 days of data per patient  *Missing Data:* n/a | Group comparisons: chi-square, independent t-tests, Mann–Whitney  Correlations: Spearman's, Pearson's, logistic regression | HDRS, BRMS | N = 38 (19 BDI, 19 HC) |
| Kaczmarek-Majer et al (2024) [66] | **Speech**: The study showed that prosodic, spectral, and voice quality parameters are all valid  markers in assessing the severity of manic and depressive symptoms. The greater the severity of mania in males, the louder and higher the tone of voice. For females, the greater the severity of mania, the quieter and lower the tone of voice. | *Device:* Smartphone (BDmon app)  *Measurements:* speech during all phone calls  *Data Characteristics:* phone calls every fortnight, used on average of 208 days  *Missing Data:* n/a | Generalized mixed-effect model | HDRS, YMRS (every 3 months, and additionally when a mood change was suspected during fortnightly phone calls) | N = 51, mix of inpatient and outpatient |
| Karam et al (2014) [69] | **Speech**: Classified mania (AUC=0.81) and depression (AUC=0.67) solely using speech colleced during clinical evaluations. | *Device:* Smartphone (custom app)  *Measurements:* records outgoing speech at 8KhZ  *Data Characteristics:* 6-31 weeks of weekly calls  *Missing Data: n/a* | Support Vector Machines | HDRS, YMRS (weekly over phone) | N = 6, outpatient |
| Kim et al (2024) [43] | **Multi-Marker Classification (Sleep, Physical Activity, Heart Rate, Light Exposure)**: Use deep learning to predict major depressive episodes 7 days in advance across MDD and BD. Shifts in dim light melatonin onset (DLMO) was the most predictive feature. LSTM and GRU acheived a recall of 0.748 and 0.767 respectively, and an AUROC of 0.581 and 0.565 respectively. | *Device:* Wearable (Fitbit Charge), Smartphone (Android)  *Measurements:* sleep, steps, heart rate, ambient light  *Data Characteristics:* continuously for a minimum of 30 days  *Missing Data: "*To maintain consistent data, missing values were minimized, allowing up to 15 missing values across the 34 features in the 7-day data, considering the number of patients. MinMaxScaler was applied to normalize the range of features between 0 and 1. The training consisted of two stages: missing value imputation and depressive episode prediction. Missing values were imputed through deep learning-based predictions during the first stage." - no further data was provided. | LSTM, GRU, LSTM-GRU | MADRS (every 3 months) | N = 164, (43 BD I, 65 BD II, 56 MDD), outpatient |
| Langholm et al (2023) [74] | **Multi-Marker Classification (Geolocation, Smartphone Usage):** Classification of MDD or BD had an AUC of 0.62 | *Device:* Smartphone (Android)  *Measurements:* Geolocation, accelerometer, screen-state (on/off)  *Data Characteristics:* 12-week study using MindLamp app  *Missing Data:* 14.9% missingness of passive data | Random Forest, SVM, Naive Bayes, K-means clustering | QIDS-CR (Depression), YMRS (Mania), PHQ-2, GAD-2 (3 notifications/ week) | N=116 (53 MDD, 28 BD, 35 HC), outpatient |
| Lee et al (2023) [37] | **Multi-Marker Classification (Sleep, Physical Activity, Heart Rate, Light Exposure)**: This study aims to use circadian rhythm disruptions measured through aggregated wearable data to classify upcoming depressive/manic episodes over the next 3 days. The prediction accuracies for impending major depressive episodes was 90.1% (AUC of 0.937), manic episodes was 92.6% (AUC of 0.957), and hypomanic episodes was 93% (AUC of 0.963) for the next 3 days | *Device:* Wearable (Fitbit Charge), Smartphone (Android)  *Measurements:* sleep, steps, heart rate, ambient light  *Data Characteristics:* continuously for a minimum of 30 days, average of 279.7 days  *Missing Data: "*During the data collection period, we assembled 75 506 sample days [mean (S.D.) days per participant, 279.7 (263.5)] from 270 participants after data imputation processing. The dataset for prediction modeling has 140 features (variables) plus a class label of episode type. In the dataset, 38% of the whole cells of our tabular data (=75 506 samples × 140 features) were missing and therefore compensated by the imputation procedure." | Random Forest | YMRS, MADRS (every 3 months) | N = 175, (78 BD I, 97 BD II), outpatient |
| Lee et al (2025) [31] | **Geolocation**: GPS-derived mobility features and their Fourier power spectra differentiated BP vs MDD and tracked depressed mood. Key GPS indicators were location variance (LV), transition time (TT), and entropy. Fourier analysis showed BP had clearer periodic mobility patterns (eg, 1-, 4-, 9-day cycles), and maximum power spectra of LV and entropy differed between BP and MDD. | *Device:* Smartphone (Android)  *Measurements:* GPS location  *Data Characteristics:* 5-180 days  *Missing Data:* went from 6330 to 5177 persoon-days after excluding days with <25% data | Logistic regression | YMRS, HAMD | N = 62 (20 BD, 27 MDD, 15 HC) |
| Lim et al (2024) [55] | **Multi-Marker Classification (Sleep):** Features related to sleep and circadan rhythms enabled accurate next-day predictions for depressive, manic, and hypomanic episodes (AUCs: 0.80, 0.98, 0.95). Daily circadian phase shifts were the most significant predictors: delays linked to depressive episodes, advances to manic episodes. | *Device:* Wearable (Fitbit Charge)  *Measurements:* sleep derived 36 sleep and circadian rhythm features  *Data Characteristics:* minimum 30 days per patient, average of 267 days  *Missing Data:* not well detailed, to our knowledge only say average sleep timeseries goes from 429 days to 267 days across patients after excluding missing data | XGBoost | YMRS, MADRS (every 3 months) | N = 111, outpatient |
| Lipschitz et al (2024) [38] | **Multi-Marker Classification (Sleep, Physical Activity, Heart Rate):** Aggregated wearable data was able to classify depression (AUC=0.86) and mania (AUC=0.85) | *Device:* Wearable (Fitbit Inspire)  *Measurements:* 17 features related to sleep, activity, and HR  *Data Characteristics:* continuously for 9 months  *Missing Data:* "Full or partial data imputation was performed on 620 study days (out of 14,406 total days across all participants in the study). This translates to 4.3% of Fitbit data days (a mean of 11 days per participant). Across participants, days with imputed data ranged from 0% to 34%." "On average, participants included in the analysis were compliant with the Fitbit protocol 87.8% of weeks." | Binary Mixed Model forest | ASRM, PHQ-8 (biweekly) | N = 54, outpatient |
| Liu et al (2024) [57] | **Smartphone Usage**: Higher smartphone keyboard backspace rates are associated with symptoms of mania and depression. Individuals were grouped into low, medium, and high backspace rate groups. The medium group had significantly higher ratings of depression, and both high and medium were significantly associated with higher ratings of mania. | Device: Smartphone (BiAffect app on Android smartphones)  *Measurements:* backspace rates  *Data Characteristics:* used as primary keyboard for 4 weeks  *Missing Data: n/a* | Bayesian Mixture Model | HDRS, YMRS (week 2 and 4) | N = 101 (83 MDD, 14 BD, 3 PDD, 1 cyclothymia), outpatient |
| Ludwig et al (2025a) [27] | **Multi-Marker (Physical Activity, Smartphone Usage, Geolocation)**: Early warning signals (EWS) derived from smartphone parameters distinguished pre-episode weeks (early/late) from euthymia for both depression and mania. Markers were autocorrelation (AR), variance (VAR), and moving averages (AVG) of specific variables. | *Device:* Smartphone (Android)  *Measurements:* GPS location, accelerometer-based physical activity, screen on/off status, and communication behavior  *Data Characteristics:* continuously up to 1 year  *Missing Data:* "Passive sensing data were available for 99 % of patient days, with 1 % (n = 152) completely missing due to phone issues" | Multilevel Logit Models | YMRS, MADRS (biweekly) | N = 29 (17 BD I, 12 BD II) |
| Ludwig et al (2025b) [28] | **Multi-Marker (Physical Activity, Smartphone Usage, Geolocation)**: Activity features showed the clearest signal for (hypo)mania, with kilometers traveled fast distinguishing early preepisode weeks (β/OR=1.0/2.7), late preepisode weeks (β/OR=1.7/5.7), first episode week (β/OR=1.1/3.1), and second episode week (β/OR=1.0/2.8) from euthymia. Smartphone socialization features were more informative for depression, with outgoing calls, missed calls, outgoing-not-reached calls, and unique conversation partners distinguishing selected depressive preepisode or episode weeks. However, effects were inconsistent across variables and time windows. | *Device:* Smartphone (movisensXS app)  *Measurements:* activity indicators (steps, minutes in vehicle/on foot/still, kilometers traveled), communicativeness indicators (outgoing calls, incoming missed calls, outgoing not reached calls, call duration, unique conversation partners), smartphone usage (display on, phone inactive)  *Data Characteristics:* 12 months of continuously collected passive smartphone sensing data  *Missing Data:* 856 out of 10214 days were labeled as missing | Linear mixed effects models | SCID-5 section A (biweekly) | N = 28 (16 BD I, 12 BD II), outpatients |
| Nardelli et al (2017) [35] | **Heart Rate:** There is a clear loss of complexity modulation of heartbeat dynamics during depressive and hypomanic states compared to euthymia. | *Device:* Wearable (Wearable system comprised of a comfortable t-shirt with integrated fabric electrodes and sensors)  *Measurements:* ECGs  *Data Characteristics:* daytime (8am-8pm), nighttime (8pm-7:30am) (2-3 times a month)  *Missing Data: n/a* | Multiscale entropy analysis | QIDS (2-3 times a month) | N = 8, inpatient |
| Ortiz et al (2025) [49] | **Multi-Marker (Physical Activity, Sleep):** 12-hr variability in sleep (balanced accuracy = 0.87) and metabolic activity (balanced accuracy = 0.89) achieved high performance in detecting hypomanic episode onset, predicting the onset with a median delay of 3 and 2.5 days respectively. | *Device:* Wearable (Oura Health Oy, Generation 2)  *Measurements:* sleep stages in 5 minute intervals, metabolic activity in 5 minute intervals  *Data Characteristics:* collected continuously for a median of 495 days  *Missing Data:* "participants used the wearable 86 % of the time on average, with a non-wear time of median (IQR) of 31.4 (33.6) days" | Time-series oscillatory mode decomposition, Hilbert transform, and spectral derivative computation followed by peak detection to determine time-frequency variability | ASRM, PHQ-9 (weekly) | N = 50, outpatients |
| Palmius et al (2017) [25] | **Geolocation**: There is a strong link between geographic movements and depression in bipolar disorder, demonstrating an optimal mean absolute error rate when predicting clinical scale scores of 3.73, and a classification accuracy of 0.849. | *Device:* Smartphone (custom app on provided smartphone)  *Measurements:* anonymized geolocation data  *Data Characteristics:* daily for an average of 8.4 weeks in euthymia, 8.6 weeks in depressive patients  *Missing Data: "*Missing data were imputed in sections where the participant was recorded at a location within 500 m either end of the missing section, and where the missing section had a length of 1) 2 h or less any time; or 2) 12 h or less after 9 PM. The missing section was filled with the mean latitude and longitude of the coordinates on either end." No further missing data was reported. | Linear regression models, a quadratic discriminant analysis classifier | QIDS (weekly) | N = 22, outpatient |
| Pan et al (2018) [70] | **Speech:** Spontaneous conversations with clinicians were used to classify manic vs euthymic states, where Support Vector Machines were superior for classification within individuals (88.56% accuracy) and Gaussian Mixture Models were superior for classification across individuals (72.27% accuracy). | *Device:* Smartphone (preloaded Samsung GALAXY Mega 6.3, calls recorded over Cloud network)  *Measurements:* clinican would have free open conversation which was recorded and transferred to a cloud database  *Data Characteristics:* recorded for 10-25 minutes twice in each mood state (manic and euthymic) in the morning in consecutive 1–2 days  *Missing Data: n/a* | Support Vector Machines, Gaussian Mixture Models | BRMS (at admission) | N = 21, inpatient |
| Stange et al (2018) [58] | **Smartphone Usage:** Instability of typing speed predicted elevated future symptoms of depression, but not mania. Passive assessments of typing instability accounted for a similar amount of variance in future mood symptoms compared to actively reported EMA mood instability. | *Device:* Smartphone (BiAffect app on Android smartphones)  *Measurements:* keyboard strokes passively collected  *Data Characteristics:* prompted semi-randomly once a day over 2 weeks  *Missing Data: n/a* | Multilevel models (using MPlus), bootstrapped mediation analysis | HDRS, YMRS (weekly) | N = 18, outpatient |
| Tanaka et al (2018) [51] | **Physical Activity**: BD participants showed high activity pattern in the morning and low activity pattern in evenings, with MDD demonstrating the opposite pattern | *Device:* Wearable (Actiwatch)  *Measurements:* Accelerometry  *Data Characteristics:* collected for 21 days  *Missing Data: n/a* | Principal Component Analysis | not specified | N = 59 (35 BD, 59 MDD), inpatient |
| Valenza et al (2014) [18] | **Heart Rate:** Personalized monitoring models to recognize mood states with a total classification accuracy up to 95.81%. | *Device:* Wearable (custom system comprised of a t-shirt with integrated fabric electrodes and sensors)  *Measurements:* ECGs  *Data Characteristics:* worn in the afternoon till morning (18hr) 3-6 times  *Missing Data: n/a* | Markov chain models | HDRS, YMRS (2-3 times a month) | N = 8, inpatient |
| Valenza et al (2016) [34] | **Heart Rate:** Personalized prediction accuracies in forecasting a mood state (euthymia/non-euthymia) at day t+1 were 69% on average, reaching values as high as 83.3%. | *Device:* Wearable (custom system comprised of a t-shirt with integrated fabric electrodes and sensors)  *Measurements:* ECGs  *Data Characteristics:* during the evening and night twice a week for 14 weeks  *Missing Data:* the longest artifact-free segment is reported per patient | Support Vector Machines | HDRS, YMRS (at admission) | N = 14, inpatient |
| Wu et al (2025) [73] | **Multi-Marker Classification (Sleep, Physical Activity, Heart Rate):** Wearable-derived digital biomarkers were used to predict depressive and manic symptom states in BD using supervised ML; interpretability analysis suggested higher resting HR, lower activity, and reduced sleep as key predictors of depressive symptoms. | *Device:* Wearable (Garmin Vivosmart 4)  *Measurements:* motor activities - steps, distance traveled, and floors climbed; sleep features - total sleep hours and stages of sleep, that is, deep sleep, light sleep, rapid eye movement sleep, and awake stages; HR features - the minimum HR of the day, the maximum HR of the day, the average HR of the day, and the average HR at rest.  *Data Characteristics:* average of 6.39 months  *Missing Data: n/a* | Logistic Regression, Decision Tree, K-Nearest Neighbors, Random Forest, AdaBoost, XGBoost | YMRS, BDI (weekly) | N = 24 |
| Zhang et al (2018) [65] | **Speech:** Established characteristic speech signals that differed between manic and euthymic groups. Specifically, the fourth formant and Linear Prediction Coefficient were significantly differed when patients progressed from manic to remission state. | *Device:* Smartphone (custom app developed by team on provided Android mobile phones)  *Measurements:* audio recording outgoing calls in a quiet indoor room  *Data Characteristics:* 25-30 min clinical phone conversations, two times  *Missing Data: n/a* | Two-sided t-test, chi-square tests | BRMS (after 2 audio collection sessions) | N = 30, outpatient |
| Zulueta et al (2018) [59] | **Smartphone Usage:** Depression scores were positively correlated with accelerometer displacement, average interkey delay, session count, and autocorrect rate. For mania, accelerometer displacement is positively correlated and backspace rate is negatively correlated | Device: Smartphone (BiAffect app on Android smartphones)  Measurements: keyboard strokes passively collected  *Data Characteristics:* passively collected over 8 weeks  *Missing Data: "*missing data were handled with pairwise deletion" | Mixed-effects regression model | HDRS, YMRS (weekly) | N = 9 (5 BD I, 4 BD II), outpatient |

**Table S2:** PROBAST Assessment

| **Author (Year)** | **Participants** | **Predictors** | **Outcome** | **Analysis** | **Overall** |
| --- | --- | --- | --- | --- | --- |
| Valenza et al (2016) [34] | Low | Low | Low | High | High |
| Ortiz et al (2025) [49] | Low | Low | Low | High | High |
| Lim et al (2024) [55] | Low | Low | Low | High | High |
| Palmius et al (2017) [25] | Low | Low | Low | High | High |
| Anmella et al (2023) [20] | Low | Low | Low | High | High |
| Cote-Allard et al (2022) [21] | Low | Low | Low | High | High |
| Cho et al (2019) [36] | Low | Low | Low | High | High |
| Lipschitz et al (2024) [38] | Low | Low | Low | High | High |
| Ji et al (2024) [67] | Low | Low | Low | High | High |
| Faurholt-Jepsen et al (2016a) [23] | Low | Low | Low | High | High |
| Karam et al (2014) [69] | Low | Low | Low | High | High |
| Pan et al (2018) [70] | Low | Low | Low | High | High |
| Cao et al (2017) [60] | Low | Low | Low | High | High |
| Grünerbl et al (2015) [63] | Low | Low | Low | High | High |
| Kim et al (2024) [43] | Low | Low | Low | High | High |
| Ikäheimonen et al (2024) [56] | Low | Low | Low | High | High |
| Lee et al (2023) [37] | Low | Low | Low | High | High |
| Wu et al (2025) [73] | Low | Low | Low | High | High |
| Faurholt-Jepsen et al (2022b) [72] | Low | Low | Low | High | High |
| Faurholt-Jepsen et al (2024) [64] | Low | Low | Low | High | High |
| Tanaka et al (2018) [51] | Low | Low | Low | High | High |
| Faurholt-Jepsen et al (2021b) [71] | Low | Low | Low | High | High |
| Langholm et al (2023) [74] | Low | Low | Low | High | High |

**Table S3:** Newcastle-Ottawa Assessment

| **Author (Year)** | **Study Type** | **Selection** | **Comparability** | **Exposure (cc)** | **Outcome (co)** | **TOTAL** |
| --- | --- | --- | --- | --- | --- | --- |
| Esaki et al (2021a) [50] | Cohort | Representative, Exposure (2) | 2 |  | Assessment, Follow-Up, Adequately (3) | 7 |
| Gershon et al (2016) [46] | Cohort | Representative, Control, Exposure, Outcome (4) | 2 |  | Assessment, Follow-up (2) | 8 |
| Jakobsen et al (2022) [48] | Cohort | Representative, Exposure, Outcome (3) | 2 |  | Assessment (1) | 6 |
| Anmella et al (2024) [19] | Cohort | Representative, Exposure, Outcome (3) | 2 |  | Assessment (1) | 6 |
| Faurholt-Jepsen et al (2014) [22] | Cohort | Representative, Exposure, Outcome (3) | 0 |  | Assessment, Follow-Up, Adequately (3) | 6 |
| Faurholt-Jepsen et al (2021) [24] | Cohort | Representative, Control, Exposure, Outcome (4) | 0 |  | Assessment (1) | 5 |
| Faurholt-Jepsen et al (2017) [32] | Cohort | Representative, Exposure, Outcome (3) | 2 |  | Assessment, Follow-up (2) | 7 |
| Nardelli et al (2017) [35] | Cohort | Representative, Exposure (2) | 0 |  | Assessment, Follow-Up, Adequately (3) | 5 |
| Corponi et al (2024) [33] | Cohort | Representative, Exposure, Outcome (3) | 2 |  | Assessment, Follow-Up, Adequately (3) | 8 |
| Liu et al (2024) [57] | Cohort | Representative, Exposure (2) | 2 |  | Assessment, Follow-Up, Adequately (3) | 7 |
| Stange et al (2018) [58] | Cohort | Representative, Exposure (2) | 2 |  | Assessment, Follow-up (2) | 6 |
| Zulueta et al (2018) [59] | Cohort | Representative, Exposure (2) | 2 |  | Assessment, Follow-Up, Adequately (3) | 7 |
| Esaki et al (2022) [40] | Cohort | Representative, Exposure, Outcome (3) | 2 |  | Assessment, Follow-Up, Adequately (3) | 8 |
| Esaki et al (2020) [39] | Cohort | Representative, Exposure, Outcome (3) | 2 |  | Assessment, Follow-Up, Adequately (3) | 8 |
| Esaki et al (2019) [41] | Cohort | Representative, Exposure, Outcome (3) | 2 |  | Assessment, Follow-Up, Adequately (3) | 8 |
| Esaki et al (2021b) [42] | Cohort | Representative, Exposure, Outcome (3) | 2 |  | Assessment, Follow-Up, Adequately (3) | 8 |
| Faurholt-Jepsen et al (2016a) [23] | Cohort | Representative, Exposure, Outcome (3) | 2 |  | Assessment, Follow-Up, Adequately (3) | 8 |
| Beiwinkel et al (2016) [26] | Cohort | Representative, Exposure, Outcome (3) | 2 |  | Assessment, Follow-Up, Adequately (3) | 8 |
| Faurholt-Jepsen et al (2016b) [68] | Cohort | Representative, Exposure, Outcome (3) | 2 |  | Assessment, Follow-up (2) | 7 |
| Esaki et al (2023) [53] | Cohort | Representative, Exposure, Outcome (3) | 2 |  | Assessment, Follow-Up, Adequately (3) | 8 |
| Dominiak et al (2022) [62] | Cohort | Representative, Exposure, Outcome (3) | 2 |  | Assessment, Follow-Up, Adequately (3) | 8 |
| Faurholt-Jepsen et al (2015b) [61] | Cohort | Representative, Exposure, Outcome (3) | 2 |  | Assessment, Follow-Up, Adequately (3) | 8 |
| Zhang et al (2018) [65] | Cohort | Representative, Exposure, Outcome (3) | 2 |  | Assessment, Follow-Up, Adequately (3) | 8 |
| Kaczmarek-Majer et al (2024) [66] | Cohort | Representative, Exposure, Outcome (3) | 2 |  | Assessment, Follow-Up, Adequately (3) | 8 |
| Valenza et al (2014) [18] | Cohort | Representative, Exposure, Outcome (3) | 2 |  | Assessment, Follow-up (2) | 7 |
| Ebner-Priemer et al (2020) [44] | Cohort | Representative, Exposure, Outcome (3) | 2 |  | Assessment, Follow-Up, Adequately (3) | 8 |
| Esaki et al (2024) [54] | Cohort | Representative, Exposure, Outcome (3) | 2 |  | Assessment, Follow-Up, Adequately (3) | 8 |
| Clemens et al (2025) [47] | Cohort | Representative, Exposure, Outcome (3) | 1 |  | Assessment, Follow-Up, Adequately (3) | 7 |
| Faurholt-Jepsen et al (2022a) [30] | Cohort | Representative, Control, Exposure (3) | 1 |  | Assessment, Follow-Up, Adequately (3) | 7 |
| Jones et al (2005) [52] | Case-control | Representative, Control, Exposure (3) | 1 |  | Assessment (1) | 5 |
| Aledavood et al (2025) [29] | Cohort | Representative, Control, Exposure, Outcome (4) | 2 |  | Assessment, Follow-Up, Adequately (3) | 9 |
| Lee et al (2025) [31] | Cohort | Representative, Control, Exposure, Outcome (4) | 2 |  | Assessment, Follow-Up, Adequately (3) | 9 |
| Ludwig et al (2025a) [27] | Cohort | Representative, Control, Exposure, Outcome (4) | 2 |  | Assessment, Follow-Up, Adequately (3) | 9 |
| Ludwig et al (2025b) [28] | Cohort | Representative, Control, Exposure, Outcome (4) | 2 |  | Assessment, Follow-Up, Adequately (3) | 9 |

**References**

1. Grande I, Berk M, Birmaher B, Vieta E. Bipolar disorder. The Lancet. Apr 2016;387(10027):1561-1572. [doi: 10.1016/S0140-6736(15)00241-X]

2. Vieta E, Berk M, Schulze TG, et al. Bipolar disorders. Nat Rev Dis Primers. Mar 8, 2018;4(1):18008. [doi: 10.1038/nrdp.2018.8] [Medline: 29516993]

3. Judd LL, Akiskal HS, Schettler PJ, et al. The long-term natural history of the weekly symptomatic status of bipolar I disorder. Arch Gen Psychiatry. Jun 2002;59(6):530-537. [doi: 10.1001/archpsyc.59.6.530] [Medline: 12044195]

4. Judd LL, Akiskal HS, Schettler PJ, et al. A prospective investigation of the natural history of the long-term weekly symptomatic status of bipolar II disorder. Arch Gen Psychiatry. Mar 2003;60(3):261-269. [doi: 10.1001/archpsyc.60.3.261] [Medline: 12622659]

5. Simon GE, Hunkeler E, Fireman B, Lee JY, Savarino J. Risk of suicide attempt and suicide death in patients treated for bipolar disorder. Bipolar Disord. Aug 2007;9(5):526-530. [doi: 10.1111/j.1399-5618.2007.00408.x] [Medline: 17680924]

6. Plans L, Barrot C, Nieto E, et al. Association between completed suicide and bipolar disorder: a systematic review of the literature. J Affect Disord. Jan 1, 2019;242:111-122. [doi: 10.1016/j.jad.2018.08.054] [Medline: 30173059]

7. Ortiz A, Mulsant BH. Beyond step count: are we ready to use digital phenotyping to make actionable individual predictions in psychiatry? J Med Internet Res. Aug 5, 2024;26:e59826. [doi: 10.2196/59826] [Medline: 39102686]

8. Bauer M, Andreassen OA, Geddes JR, et al. Areas of uncertainties and unmet needs in bipolar disorders: clinical and research perspectives. Lancet Psychiatry. Nov 2018;5(11):930-939. [doi: 10.1016/S2215-0366(18)30253-0] [Medline: 30146246]

9. Faurholt-Jepsen M, Frost M, Ritz C, et al. Daily electronic self-monitoring in bipolar disorder using smartphones - the MONARCA I trial: a randomized, placebo-controlled, single-blind, parallel group trial. Psychol Med. Oct2015;45(13):2691-2704. [doi: 10.1017/S0033291715000410] [Medline: 26220802]

10. Faurholt-Jepsen M, Frost M, Christensen EM, Bardram JE, Vinberg M, Kessing LV. The effect of smartphone-based monitoring on illness activity in bipolar disorder: the MONARCA II randomized controlled single-blinded trial. Psychol Med. Apr 2020;50(5):838-848. [doi: 10.1017/S0033291719000710] [Medline: 30944054]

11. Peralta V, Cuesta MJ. Lack of insight in mood disorders. J Affect Disord. Apr 1998;49(1):55-58. [doi: 10.1016/s0165-0327(97)00198-5] [Medline: 9574860]

12. Saccaro LF, Amatori G, Cappelli A, Mazziotti R, Dell’Osso L, Rutigliano G. Portable technologies for digital phenotyping of bipolar disorder: a systematic review. J Affect Disord. Dec 1, 2021;295:323-338. [doi: 10.1016/j.jad.2021.08.052] [Medline: 34488086]

13. Ortiz A, Maslej MM, Husain MI, Daskalakis ZJ, Mulsant BH. Apps and gaps in bipolar disorder: a systematic review on electronic monitoring for episode prediction. J Affect Disord. Dec 1, 2021;295(1190–1200):1190-1200. [doi: 10.1016/j.jad.2021.08.140] [Medline: 34706433]

14. Page MJ, McKenzie JE, Bossuyt PM, et al. The PRISMA 2020 statement: an updated guideline for reporting systematic reviews. BMJ. Mar 29, 2021;372:n71. [doi: 10.1136/bmj.n71] [Medline: 33782057]

15. Ouzzani M, Hammady H, Fedorowicz Z, Elmagarmid A. Rayyan-a web and mobile app for systematic reviews. Syst Rev. Dec 5, 2016;5(1):210. [doi: 10.1186/s13643-016-0384-4] [Medline: 27919275]

16. Moons KGM, Wolff RF, Riley RD, et al. PROBAST: a tool to assess risk of bias and applicability of prediction model studies: explanation and elaboration. Ann Intern Med. Jan 1, 2019;170(1):W1-W33. [doi: 10.7326/M18-1377] [Medline: 30596876]

17. Wells G, Shea B, O’Connel D, et al. The newcastle-ottawa scale (NOS) for assessing the quality of nonrandomised studies in meta-analyses. Ottawa Hospital Research Institute (OHRI). 2000. URL: <https://www.ohri.ca/programs/clinical_epidemiology/oxford.asp> [Accessed 2025-02-28]

18. Valenza G, Nardelli M, Lanatà A, et al. Wearable monitoring for mood recognition in bipolar disorder based on history-dependent long-term heart rate variability analysis. IEEE J Biomed Health Inform. Sep 2014;18(5):1625-1635. [doi: 10.1109/JBHI.2013.2290382] [Medline: 24240031]

19. Anmella G, Mas A, Sanabra M, et al. Electrodermal activity in bipolar disorder: Differences between mood episodes and clinical remission using a wearable device in a real-world clinical setting. J Affect Disord. Jan 15, 2024;345:43-50. [doi: 10.1016/j.jad.2023.10.125] [Medline: 37865347]

20. Anmella G, Corponi F, Li BM, et al. Exploring digital biomarkers of illness activity in mood episodes: hypotheses generating and model development study. JMIR Mhealth Uhealth. May 4, 2023;11:e45405. [doi: 10.2196/45405] [Medline: 36939345]

21. Cote-Allard U, Jakobsen P, Stautland A, et al. Long–short ensemble network for bipolar manic-euthymic state recognition based on wrist-worn sensors. IEEE Pervasive Comput. 2022;21(2):20-31. [doi: 10.1109/MPRV.2022.3155728]

22. Faurholt-Jepsen M, Frost M, Vinberg M, Christensen EM, Bardram JE, Kessing LV. Smartphone data as objective measures of bipolar disorder symptoms. Psychiatry Res. Jun 30, 2014;217(1-2):124-127. [doi: 10.1016/j.psychres.2014.03.009] [Medline: 24679993]

23. Faurholt-Jepsen M, Vinberg M, Frost M, et al. Behavioral activities collected through smartphones and the association with illness activity in bipolar disorder. Int J Methods Psychiatr Res. Dec 2016;25(4):309-323. [doi: 10.1002/mpr.1502] [Medline: 27038019]

24. Faurholt-Jepsen M, Busk J, Vinberg M, et al. Daily mobility patterns in patients with bipolar disorder and healthy individuals. J Affect Disord. Jan 1, 2021;278:413-422. [doi: 10.1016/j.jad.2020.09.087] [Medline: 33010566]

25. Palmius N, Tsanas A, Saunders KEA, et al. Detecting bipolar depression from geographic location data. IEEE Trans Biomed Eng. Aug 2017;64(8):1761-1771. [doi: 10.1109/TBME.2016.2611862] [Medline: 28113247]

26. Beiwinkel T, Kindermann S, Maier A, et al. Using smartphones to monitor bipolar disorder symptoms: a pilot study. JMIR Ment Health. Jan 6, 2016;3(1):e2. [doi: 10.2196/mental.4560] [Medline: 26740354]

27. Ludwig VM, Bittendorf CA, Reinhard I, et al. Early warning signals of bipolar relapse: investigating critical slowing down in smartphone data. J Affect Disord. Dec 15, 2025;391:119972. [doi: 10.1016/j.jad.2025.119972] [Medline: 40738339]

28. Ludwig VM, Bittendorf CA, Reinhard I, et al. Predicting depressive and manic episodes in patients with bipolar disorder using statistical process control methods on passive sensing data. J Psychopathol Clin Sci. Nov2025;134(8):971-981. [doi: 10.1037/abn0001002] [Medline: 40689911]

29. Aledavood T, Luong N, Baryshnikov I, et al. Multimodal digital phenotyping study in patients with major depressive episodes and healthy controls (mobile monitoring of mood): observational longitudinal study. JMIR Ment Health. Feb 21, 2025;12:e63622. [doi: 10.2196/63622] [Medline: 39984168]

30. Faurholt-Jepsen M, Busk J, Rohani DA, et al. Differences in mobility patterns according to machine learning models in patients with bipolar disorder and patients with unipolar disorder. J Affect Disord. Jun 1, 2022;306:246-253. [doi: 10.1016/j.jad.2022.03.054] [Medline: 35339568]

31. Lee TY, Chen CH, Liu CM, et al. Fourier transform analysis of GPS-derived mobility patterns for diagnosis and mood monitoring of bipolar and major depressive disorders: prospective study. J Med Internet Res. Jul 15, 2025;27:e71658. [doi: 10.2196/71658] [Medline: 40702785]

32. Faurholt-Jepsen M, Brage S, Kessing LV, Munkholm K. State-related differences in heart rate variability in bipolar disorder. J Psychiatr Res. Jan 2017;84:169-173. [doi: 10.1016/j.jpsychires.2016.10.005] [Medline: 27743529]

33. Corponi F, Li BM, Anmella G, et al. A Bayesian analysis of heart rate variability changes over acute episodes of bipolar disorder. Npj Ment Health Res. Oct 3, 2024;3(1):44. [doi: 10.1038/s44184-024-00090-x] [Medline: 39363115]

34. Valenza G, Nardelli M, Lanata’ A, et al. Predicting mood changes in bipolar disorder through heartbeat nonlinear dynamics. IEEE J Biomed Health Inform. Jul 2016;20(4):1034-1043. [doi: 10.1109/JBHI.2016.2554546] [Medline: 28113920]

35. Nardelli M, Lanata A, Bertschy G, Scilingo EP, Valenza G. Heartbeat complexity modulation in bipolar disorder during daytime and nighttime. Sci Rep. Dec 20, 2017;7(1):17920. [doi: 10.1038/s41598-017-18036-z] [Medline: 29263393]

36. Cho CH, Lee T, Kim MG, In HP, Kim L, Lee HJ. Mood prediction of patients with mood disorders by machine learning using passive digital phenotypes based on the circadian rhythm: prospective observational cohort study. J Med Internet Res. Apr 17, 2019;21(4):e11029. [doi: 10.2196/11029] [Medline: 30994461]

37. Lee HJ, Cho CH, Lee T, et al. Prediction of impending mood episode recurrence using real-time digital phenotypes in major depression and bipolar disorders in South Korea: a prospective nationwide cohort study. Psychol Med. Sep2023;53(12):5636-5644. [doi: 10.1017/S0033291722002847] [Medline: 36146953]

38. Lipschitz JM, Lin S, Saghafian S, Pike CK, Burdick KE. Digital phenotyping in bipolar disorder: using longitudinal Fitbit data and personalized machine learning to predict mood symptomatology. Acta Psychiatr Scand. Mar2025;151(3):434-447. [doi: 10.1111/acps.13765] [Medline: 39397313]

39. Esaki Y, Obayashi K, Saeki K, Fujita K, Iwata N, Kitajima T. Association between light exposure at night and manic symptoms in bipolar disorder: cross-sectional analysis of the APPLE cohort. Chronobiol Int. Jun 2020;37(6):887-896. [doi: 10.1080/07420528.2020.1746799] [Medline: 32238002]

40. Esaki Y, Obayashi K, Saeki K, Fujita K, Iwata N, Kitajima T. Effect of nighttime bedroom light exposure on mood episode relapses in bipolar disorder. Acta Psychiatr Scand. Jul 2022;146(1):64-73. [doi: 10.1111/acps.13422] [Medline: 35253206]

41. Esaki Y, Kitajima T, Obayashi K, Saeki K, Fujita K, Iwata N. Daytime light exposure in daily life and depressive symptoms in bipolar disorder: a cross-sectional analysis in the APPLE cohort. J Psychiatr Res. Sep 2019;116:151-156. [doi: 10.1016/j.jpsychires.2019.06.010] [Medline: 31247358]

42. Esaki Y, Obayashi K, Saeki K, Fujita K, Iwata N, Kitajima T. Preventive effect of morning light exposure on relapse into depressive episode in bipolar disorder. Acta Psychiatr Scand. Apr 2021;143(4):328-338. [doi: 10.1111/acps.13287] [Medline: 33587769]

43. Kim B, Chae M, Kim Y, et al. Early prediction of depressive episodes in mood disorders using circadian rhythm indicators and deep learning. Presented at: 2024 IEEE International Conference on Bioinformatics and Biomedicine (BIBM); Dec 3-6, 2024:6411-6418; Lisbon, Portugal. [doi: 10.1109/BIBM62325.2024.10822654]

44. Ebner-Priemer UW, Mühlbauer E, Neubauer AB, et al. Digital phenotyping: towards replicable findings with comprehensive assessments and integrative models in bipolar disorders. Int J Bipolar Disord. Nov 17, 2020;8(1):35. [doi: 10.1186/s40345-020-00210-4] [Medline: 33211262]

45. Faurholt-Jepsen M, Brage S, Vinberg M, Kessing LV. State-related differences in the level of psychomotor activity in patients with bipolar disorder - continuous heart rate and movement monitoring. Psychiatry Res. Mar 30, 2016;237:166-174. [doi: 10.1016/j.psychres.2016.01.047] [Medline: 26832835]

46. Gershon A, Ram N, Johnson SL, Harvey AG, Zeitzer JM. Daily actigraphy profiles distinguish depressive and interepisode states in bipolar disorder. Clin Psychol Sci. Jul 2016;4(4):641-650. [doi: 10.1177/2167702615604613] [Medline: 27642544]

47. Clemens J, Mühlbauer E, Reinhard I, et al. Circadian rhythm parameters differentiate euthymic, manic and depressive mood states in bipolar disorders - an explorative pilot study. Int J Bipolar Disord. Oct 27, 2025;13(1):30. [doi: 10.1186/s40345-025-00396-5] [Medline: 41145890]

48. Jakobsen P, Stautland A, Riegler MA, et al. Complexity and variability analyses of motor activity distinguish mood states in bipolar disorder. PLoS One. 2022;17(1):e0262232. [doi: 10.1371/journal.pone.0262232] [Medline: 35061801]

49. Ortiz A, Halabi R, Alda M, et al. Day-to-day variability in sleep and activity predict the onset of a hypomanic episode in patients with bipolar disorder. J Affect Disord. Apr 1, 2025;374:75-83. [doi: 10.1016/j.jad.2025.01.026] [Medline: 39793618]

50. Esaki Y, Obayashi K, Saeki K, Fujita K, Iwata N, Kitajima T. Association between circadian activity rhythms and mood episode relapse in bipolar disorder: a 12-month prospective cohort study. Transl Psychiatry. Oct 13, 2021;11(1):525. [doi: 10.1038/s41398-021-01652-9] [Medline: 34645802]

51. Tanaka T, Kokubo K, Iwasa K, Sawa K, Yamada N, Komori M. Intraday activity levels may better reflect the differences between major depressive disorder and bipolar disorder than average daily activity levels. Front Psychol. 2018;9:2314. [doi: 10.3389/fpsyg.2018.02314] [Medline: 30581399]

52. Jones SH, Hare DJ, Evershed K. Actigraphic assessment of circadian activity and sleep patterns in bipolar disorder. Bipolar Disord. Apr 2005;7(2):176-186. [doi: 10.1111/j.1399-5618.2005.00187.x] [Medline: 15762859]

53. Esaki Y, Obayashi K, Saeki K, Fujita K, Iwata N, Kitajima T. Circadian variability of objective sleep measures predicts the relapse of a mood episode in bipolar disorder: findings from the APPLE cohort. Psychiatry Clin Neurosci. Aug 2023;77(8):442-448. [doi: 10.1111/pcn.13556] [Medline: 37092883]

54. Esaki Y, Obayashi K, Saeki K, Fujita K, Iwata N, Kitajima T. Daytime napping and depressive symptoms in bipolar disorder: a cross-sectional analysis of the APPLE cohort. Sleep Med. Dec 2024;124:688-694. [doi: 10.1016/j.sleep.2024.11.006] [Medline: 39536529]

55. Lim D, Jeong J, Song YM, et al. Accurately predicting mood episodes in mood disorder patients using wearable sleep and circadian rhythm features. NPJ Digit Med. Nov 18, 2024;7(1):324. [doi: 10.1038/s41746-024-01333-z] [Medline: 39557997]

56. Ikäheimonen A, Luong N, Baryshnikov I, et al. Predicting and monitoring symptoms in patients diagnosed with depression using smartphone data: observational study. J Med Internet Res. Dec 3, 2024;26(1):e56874. [doi: 10.2196/56874] [Medline: 39626241]

57. Liu Q, Ning E, Ross MK, et al. Digital phenotypes of mobile keyboard backspace rates and their associations with symptoms of mood disorder: algorithm development and validation. J Med Internet Res. Oct 29, 2024;26:e51269. [doi: 10.2196/51269] [Medline: 39471368]

58. Stange JP, Zulueta J, Langenecker SA, et al. Let your fingers do the talking: passive typing instability predicts future mood outcomes. Bipolar Disord. May 2018;20(3):285-288. [doi: 10.1111/bdi.12637] [Medline: 29516666]

59. Zulueta J, Piscitello A, Rasic M, et al. Predicting mood disturbance severity with mobile phone keystroke metadata: a biaffect digital phenotyping study. J Med Internet Res. Jul 20, 2018;20(7):e241. [doi: 10.2196/jmir.9775] [Medline: 30030209]

60. Cao B, Zheng L, Zhang C, et al. DeepMood: modeling mobile phone typing dynamics for mood detection. Presented at: Proceedings of the 23rd ACM SIGKDD International Conference on Knowledge Discovery and Data Mining; Aug13-17, 2017:747-755; Halifax NS, Canada. [doi: 10.1145/3097983.3098086]

61. Faurholt‐Jepsen M, Vinberg M, Frost M, Christensen EM, Bardram JE, Kessing LV. Smartphone data as an electronic biomarker of illness activity in bipolar disorder. Bipolar Disord. Nov 2015;17(7):715-728. [doi: 10.1111/bdi.12332]

62. Dominiak M, Kaczmarek-Majer K, Antosik-Wójcińska AZ, et al. Behavioral and self-reported data collected from smartphones for the assessment of depressive and manic symptoms in patients with bipolar disorder: prospective observational study. J Med Internet Res. Jan 19, 2022;24(1):e28647. [doi: 10.2196/28647] [Medline: 34874015]

63. Grünerbl A, Muaremi A, Osmani V, et al. Smartphone-based recognition of states and state changes in bipolar disorder patients. IEEE J Biomed Health Inform. Jan 2015;19(1):140-148. [doi: 10.1109/JBHI.2014.2343154] [Medline: 25073181]

64. Faurholt-Jepsen M, Rohani DA, Busk J, et al. Using digital phenotyping to classify bipolar disorder and unipolar disorder – exploratory findings using machine learning models. Eur Neuropsychopharmacol. Apr 2024;81:12-19. [doi: 10.1016/j.euroneuro.2024.01.003] [Medline: 38310716]

65. Zhang J, Pan Z, Gui C, et al. Analysis on speech signal features of manic patients. J Psychiatr Res. Mar2018;98:59-63. [doi: 10.1016/j.jpsychires.2017.12.012] [Medline: 29291581]

66. Kaczmarek-Majer K, Dominiak M, Antosik AZ, et al. Acoustic features from speech as markers of depressive and manic symptoms in bipolar disorder: a prospective study. Acta Psychiatr Scand. Mar 2025;151(3):358-374. [doi: 10.1111/acps.13735] [Medline: 39118422]

67. Ji J, Dong W, Li J, et al. Depressive and mania mood state detection through voice as a biomarker using machine learning. Front Neurol. 2024;15:1394210. [doi: 10.3389/fneur.2024.1394210] [Medline: 39026579]

68. Faurholt-Jepsen M, Busk J, Frost M, et al. Voice analysis as an objective state marker in bipolar disorder. Transl Psychiatry. Jul 19, 2016;6(7):e856-e856. [doi: 10.1038/tp.2016.123] [Medline: 27434490]

69. Karam ZN, Provost EM, Singh S, et al. Ecologically valid long-term mood monitoring of individuals with bipolar disorder using speech. Presented at: ICASSP 2014 - 2014 IEEE International Conference on Acoustics, Speech and Signal Processing (ICASSP); May 4-9, 2014:4858-4862; Florence, Italy. [doi: 10.1109/ICASSP.2014.6854525]

70. Pan Z, Gui C, Zhang J, Zhu J, Cui D. Detecting manic state of bipolar disorder based on support vector machine and Gaussian mixture model using spontaneous speech. Psychiatry Investig. Jul 2018;15(7):695-700. [doi: 10.30773/pi.2017.12.15] [Medline: 29969852]

71. Faurholt-Jepsen M, Rohani DA, Busk J, Vinberg M, Bardram JE, Kessing LV. Voice analyses using smartphone-based data in patients with bipolar disorder, unaffected relatives and healthy control individuals, and during different affective states. Int J Bipolar Disord. Dec 1, 2021;9(1):38. [doi: 10.1186/s40345-021-00243-3] [Medline: 34850296]

72. Faurholt-Jepsen M, Rohani DA, Busk J, et al. Discriminating between patients with unipolar disorder, bipolar disorder, and healthy control individuals based on voice features collected from naturalistic smartphone calls. Acta Psychiatr Scand. Mar 2022;145(3):255-267. [doi: 10.1111/acps.13391] [Medline: 34923626]

73. Wu CT, Hsieh MH, Chen IM, et al. Using wearable device and machine learning to predict mood symptoms in bipolar disorder: development and usability study. JMIR Med Inform. Sep 16, 2025;13:e66277. [doi: 10.2196/66277] [Medline: 40957006]

74. Langholm C, Breitinger S, Gray L, et al. Classifying and clustering mood disorder patients using smartphone data from a feasibility study. NPJ Digit Med. Dec 21, 2023;6(1):238. [doi: 10.1038/s41746-023-00977-7] [Medline: 38129571]

75. Cho CH, Ahn YM, Kim SJ, et al. Design and Methods of the Mood Disorder Cohort Research Consortium (MDCRC) study. Psychiatry Investig. Jan 2017;14(1):100-106. [doi: 10.4306/pi.2017.14.1.100] [Medline: 28096882]

76. Diagnostic and Statistical Manual of Mental Disorders. 5th ed. American Psychiatric Publishing; 2013:139-176. [doi: 10.1176/appi.books.9780890425596] ISBN: 9780890425596

77. Takaesu Y. Circadian rhythm in bipolar disorder: a review of the literature. Psychiatry Clin Neurosci. Sep2018;72(9):673-682. [doi: 10.1111/pcn.12688] [Medline: 29869403]

78. Thorell LH, Kjellman BF, d’Elia G. Electrodermal activity in antidepressant medicated and unmedicated depressive patients and in matched healthy subjects. Acta Psychiatr Scand. Dec 1987;76(6):684-692. [doi: 10.1111/j.1600-0447.1987.tb02940.x] [Medline: 3442260]

79. Provenza NR, Sheth SA, Dastin-van Rijn EM, et al. Long-term ecological assessment of intracranial electrophysiology synchronized to behavioral markers in obsessive-compulsive disorder. Nat Med. Dec2021;27(12):2154-2164. [doi: 10.1038/s41591-021-01550-z] [Medline: 34887577]

80. Abd-Alrazaq A, AlSaad R, Shuweihdi F, Ahmed A, Aziz S, Sheikh J. Systematic review and meta-analysis of performance of wearable artificial intelligence in detecting and predicting depression. NPJ Digit Med. May 5, 2023;6(1):84. [doi: 10.1038/s41746-023-00828-5] [Medline: 37147384]

81. Woll S, Birkenmaier D, Biri G, et al. Applying AI in the context of the association between device-based assessment of physical activity and mental health: systematic review. JMIR Mhealth Uhealth. Mar 6, 2025;13:e59660. [doi: 10.2196/59660] [Medline: 40053765]

82. Bartoli F, Cavaleri D, Crocamo C. Artificial intelligence and bipolar disorder: applications of machine learning models for diagnosis, treatment, and outcome prediction. Alpha Psychiatry. Oct 2025;26(5):44494. [doi: 10.31083/AP44494] [Medline: 41209509]

83. Sempionatto JR, Lasalde-Ramírez JA, Mahato K, Wang J, Gao W. Wearable chemical sensors for biomarker discovery in the omics era. Nat Rev Chem. Dec 2022;6(12):899-915. [doi: 10.1038/s41570-022-00439-w] [Medline: 37117704]

84. Provenza NR, Reddy S, Allam AK, et al. Disruption of neural periodicity predicts clinical response after deep brain stimulation for obsessive-compulsive disorder. Nat Med. Oct 2024;30(10):3004-3014. [doi: 10.1038/s41591-024-03125-0] [Medline: 38997607]

85. Matcham F, Barattieri di San Pietro C, Bulgari V, et al. Remote assessment of disease and relapse in major depressive disorder (RADAR-MDD): a multi-centre prospective cohort study protocol. BMC Psychiatry. Feb 18, 2019;19(1):72. [doi: 10.1186/s12888-019-2049-z] [Medline: 30777041]

86. Hurley ME, Sonig A, Herrington J, et al. Ethical considerations for integrating multimodal computer perception and neurotechnology. Front Hum Neurosci. 2024;18:1332451. [doi: 10.3389/fnhum.2024.1332451] [Medline: 38435745]

87. Kostick-Quenet KM, Herrington J, Storch EA. Personalized roadmaps for returning results from digital phenotyping. Am J Bioeth. Feb 2024;24(2):102-105. [doi: 10.1080/15265161.2023.2296454] [Medline: 38295237]

88. Martinez-Martin N, Insel TR, Dagum P, Greely HT, Cho MK. Data mining for health: staking out the ethical territory of digital phenotyping. NPJ Digital Med. Dec 19, 2018;1(1):1-5. [doi: 10.1038/s41746-018-0075-8]

89. Büscher R, Winkler T, Mocellin J, et al. A systematic review on passive sensing for the prediction of suicidal thoughts and behaviors. NPJ Mental Health Res. 2024;3(1). [doi: 10.1038/s44184-024-00089-4]

90. Bladon S, Eisner E, Bucci S, et al. A systematic review of passive data for remote monitoring in psychosis and schizophrenia. NPJ Digit Med. Jan 27, 2025;8(1):62. [doi: 10.1038/s41746-025-01451-2] [Medline: 39870797]

91. Schyvens AM, Peters B, Van Oost NC, et al. A performance validation of six commercial wrist-worn wearable sleep-tracking devices for sleep stage scoring compared to polysomnography. Sleep Adv. Apr 2025;6(2):zpaf021. [doi: 10.1093/sleepadvances/zpaf021] [Medline: 40303381]
